# Structure specific DNA recognition by the SLX1–SLX4 endonuclease complex

**DOI:** 10.1093/nar/gkab542

**Published:** 2021-06-28

**Authors:** Xiang Xu, Mingzhu Wang, Jixue Sun, Zhenyu Yu, Guohong Li, Na Yang, Rui-Ming Xu

**Affiliations:** State Key Laboratory of Medicinal Chemical Biology, College of Pharmacy and Key Laboratory of Medical Data Analysis and Statistical Research of Tianjin, Nankai University, Tianjin 300353, China; National Laboratory of Biomacromolecules, CAS Center for Excellence in Biomacromolecules, Institute of Biophysics, Chinese Academy of Sciences, Beijing 100101, China; Institutes of Physical Science and Information Technology, Anhui University, Hefei 230601, Anhui, China; State Key Laboratory of Medicinal Chemical Biology, College of Pharmacy and Key Laboratory of Medical Data Analysis and Statistical Research of Tianjin, Nankai University, Tianjin 300353, China; National Laboratory of Biomacromolecules, CAS Center for Excellence in Biomacromolecules, Institute of Biophysics, Chinese Academy of Sciences, Beijing 100101, China; National Laboratory of Biomacromolecules, CAS Center for Excellence in Biomacromolecules, Institute of Biophysics, Chinese Academy of Sciences, Beijing 100101, China; School of Life Science, University of Chinese Academy of Sciences, Beijing 100049, China; State Key Laboratory of Medicinal Chemical Biology, College of Pharmacy and Key Laboratory of Medical Data Analysis and Statistical Research of Tianjin, Nankai University, Tianjin 300353, China; National Laboratory of Biomacromolecules, CAS Center for Excellence in Biomacromolecules, Institute of Biophysics, Chinese Academy of Sciences, Beijing 100101, China; School of Life Science, University of Chinese Academy of Sciences, Beijing 100049, China

## Abstract

The SLX1–SLX4 structure-specific endonuclease complex is involved in processing diverse DNA damage intermediates, including resolution of Holliday junctions, collapse of stalled replication forks and removal of DNA flaps. The nuclease subunit SLX1 is inactive on its own, but become activated upon binding to SLX4 via its conserved C-terminal domain (CCD). Yet, how the SLX1–SLX4 complex recognizes specific DNA structure and chooses cleavage sites remains unknown. Here we show, through a combination of structural, biochemical and computational analyses, that the SAP domain of SLX4 is critical for efficient and accurate processing of 5′-flap DNA. It binds the minor groove of DNA about one turn away from the flap junction, and the 5′-flap is implicated in binding the core domain of SLX1. This binding mode accounts for specific recognition of 5′-flap DNA and specification of cleavage site by the SLX1–SLX4 complex.

## INTRODUCTION

The SLX1–SLX4 complex was originally discovered in yeast synthetic lethal screens designed to isolate proteins redundant with the Sgs1 helicase, which is important for maintenance of genome stability (1–3). Biochemical characterizations revealed that SLX1 is a highly effective structure-specific endonuclease cleaving a variety of branched DNAs including Holliday junctions (HJs), single-Y, as well as 5′-flap DNA, when forming a complex with the scaffolding protein SLX4 (4–7). In addition to its role in resolving HJs, the SLX1–SLX4 complex is also involved in the collapse of stalled replication forks and maintenance of genome integrity of ribosomal loci (1,8–11). SLX1 has also been implicated in the processing of the 5′-flap during interstrand crosslink repair, telomere maintenance and nucleotide excision repair during meiosis (12–16).

**Figure 1.**
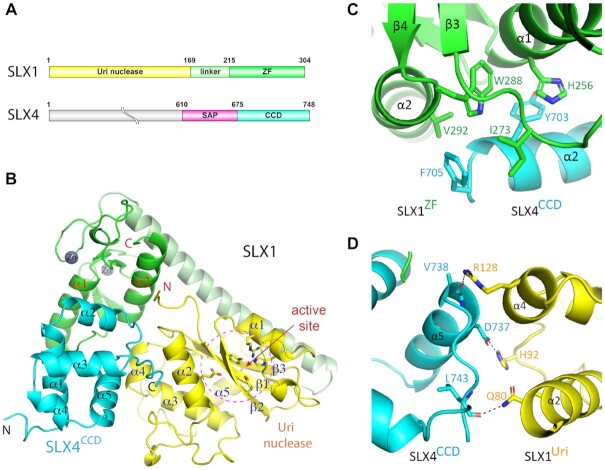
Structure of the yeast SLX1–SLX4 complex. (**A**) Schematic drawing of domain structures of yeast SLX1 and SLX4 proteins. (**B**) 1.45 Å structure of the SLX1–SLX4^CCD^ complex shown in a cartoon representation, with the Uri/GIY-YIG) domain, zinc finger (ZF) domain and the linker helix of SLX1 colored, yellow, green and pale green, respectively, and the SLX4 CCD domain is colored cyan. α-helices and β-strands in each domain are numbered consecutively and labeled. The grey spheres indicate zinc ions in the ZF domain. The conserved residues in the catalytic active site, which is indicated by dashed-line circle, are shown in a stick representation. (**C**) Mostly hydrophobic interaction between the ZF domain of SLX1 and the CCD domain of SLX4, and involved residues are displayed as sticks. (**D**) Chiefly polar interaction mediates packing of the Uri domain of SLX1 and the CCD domain of SLX4. Dashed lines indicate intermolecular hydrogen bonds between the involved residues.

SLX1 is an evolutionarily conserved protein belonging to the GIY-YIG family of nucleases. It contains two clearly identifiable domains, an N-terminal GIY-YIG nuclease domain (also called Uri domain) and a C-terminal Zinc-finger domain (Figure 1A). The GIY-YIG domain is characterized by a conserved signature sequence motif containing ‘Gly-Ile-Tyr’ and ‘Tyr-Ile-Gly’ triplets, which are found in many homing endonucleases (17). The C-terminal Zinc-finger domain of SLX1 is believed to be involved in protein-protein interactions. In comparison, SLX4 is a multidomain protein less well conserved than SLX1. Nevertheless, SLX4 proteins known to date all contain a SAF-A/B, Acinus, and PIAS (SAP) domain, followed by a conserved C-terminal domain (CCD). The CCD domain is a globular α-helical module involved in interaction with SLX1, while the SAP domain is predicted to contain a pair of α-helices suggested to bind DNA (18–20) (Figure 1A). It is reported that the SAP domain of SLX4 recruits the MUS81-EME1 3′-flap endonuclease complex in human cells, allowing the SLX1–SLX4 and MUS81–EME1 complexes to form a SLX-MUS holoenzyme that directs a distinct pathway of HJ resolution (6,21). Other reported functions of SLX4 include involvements in recruiting various proteins in diverse types of DNA processing, such as the MSH2-MSH3 mismatch-repair complex, the XPF-ERCC1 nucleotide excision-repair nuclease complex, and telomeric proteins TRF2, RAP1 and PLK1 kinase (7,12,22–25).

SLX1 alone has a very weak nuclease activity, but the binding of SLX4 greatly stimulates its enzymatic activity (4–7). The crystal structure of *Candida glabrata* SLX1 (*Cg*SLX1) shows that it forms a stable homodimer, and the dimerization blocks the active site of SLX1. Structural and biochemical analyses revealed that the binding of the CCD domain of SLX4 (*Cg*SLX4^CCD^) to the Zinc-finger domain of *Cg*SLX1 makes the active site of *Cg*SLX1 accessible to the substrate DNA (26). A structure of *Tribulus terrestris* (*Tt*) SLX1–SLX4^CCD^ in complex with DNA was reported recently (27). However, the DNA adopted an unexpected structure and was bound in an area distinct from the catalytic active site or familiar DNA binding regions in Uri domain nucleases. Thus, the mechanisms by which the SLX1–SLX4 complex recognizes DNA structure and specifies cleavage sites remain unclear. To address these important mechanistic questions, we have determined the crystal structures of a *Saccharomyces cerevisiae* SLX1–SLX4 complex in the absence and presence of a 5′-flap DNA. Besides the catalytic core of SLX1, the SAP domain of SLX4 is found to play an important role in DNA recognition and cleavage site specification. Furthermore, our molecular modeling and biochemical analyses suggest that a positive charged surface area of the SLX1 Uri domain participates in the positioning of 5′-flap DNA for effective cleavage.

## MATERIALS AND METHODS

### Protein expression and purification

cDNAs encoding *S. cerevisiae* SLX4 fragments (F1/SLX4^SAP+CCD^, a.a. 610–748; F2, a.a. 619–748; F3, a.a. 641–748; F4, 666–748; F5/SLX4^CCD^, a.a. 675–748) and full-length SLX1, or amino acid substitution mutants of which, were amplified by PCR and cloned into a pCDF-Duet vector (Novagen) at MCS I and MCS II between the NdeI-EcoRV and BamHI-SalI restriction sites, respectively. The bicistronic plasmid was transformed into the BL21 (DE3) strain of *Escherichia coli* for coexpression of the binary complex consisting of full-length SLX1 and a 6× his-tagged SLX4 fragment. Bacterial cultures were first grown at 37°C in LB medium to OD_600_ ∼ 0.8-1.0, followed by induction of protein production with 0.25 mM isopropyl β-D-1-thiogalactopyranoside (IPTG) at 16°C for 18 h. Cells were harvested by centrifugation and lysed by sonication in the lysis buffer (20 mM Tris, pH 8.0, 500 mM NaCl), followed by removal of cellular debris by centrifugation. The supernatant was incubated with Ni-NTA chelating beads (Qiagen), washed with the lysis buffer, and the bound proteins were eluted with the elution buffer (lysis buffer+500 mM imidazole). Subsequently, the eluted sample was dialyzed against a buffer of 20 mM Tris, pH 8.0, 125 mM NaCl, and loaded onto a HiTrap Q column (GE Healthcare) pre-equilibrated with the buffer. The protein complex was eluted by sodium chloride gradient, pooled and concentrated before further purification through a Superdex 75 size-exclusion column (GE Healthcare) in a buffer containing 20 mM Tris, pH 8.0 and 150 mM NaCl. We typically obtain 1–3 mg of purified SLX1–SLX4 complexes from 10 L of *E. coli* culture after the three-step purification.

### DNA oligos

All chemically synthesized DNA oligos were purchased in PAGE-purified grade from Sangon Biotech (Beijing). 5′-flap or HJ DNAs were prepared by mixing respective oligos in 20 mM Tris, pH 8.0, 150 mM NaCl, and 1 mM MgCl_2_ at equal molar ratio. The mixture was heated at 95°C for 5 min and annealed by slowly cooling down to 25°C in 5 h (sequences of DNA are shown in Supplementary Table S1).

### Crystallization and structure determination

All crystals were grown by the hanging-drop vapor diffusion method. Diffracting crystals of the SLX1–SLX4^SAP+CCD^ complex grew in a buffer containing 0.2 M potassium sodium tartrate tetrahydrate, 0.1 M sodium cacodylate, pH 6.0, 20% PEG 3350 and 0.2 M NDSB-201 at 16°C with a protein concentration of ∼3 mg/ml. Crystals of the SLX1–SLX4^CCD^ complex were grown in a buffer containing 0.2 M ammonium sulfate, 0.1 M Tris, pH 8.5 and 20% PEG 3350 at 16°C with a protein concentration of ∼10 mg/ml. The SLX1–SLX4^SAP+CCD^-5'-flap DNA complex was prepared by mixing the inactive SLX1^Y17F^-SLX4^SAP+CCD^ mutant complex with pre-annealed 5′-flap DNA at a 1:1 molar ratio, with the final protein concentration at ∼7 mg/ml. Crystals were grown in a buffer containing 0.1 M sodium cacodylate, pH 6.0, 12% PEG 1500 and 0.1 M TCEP hydrochloride at 4°C.

All X-ray diffraction data were collected at beamline BL17U of Shanghai Synchrotron Radiation Facility (SSRF) using an ADSC Q315r detector. Data collection was carried out at 100 K in a condition with 10% glycerol added to the crystallization solution, and the data were processed using HKL2000 (28). The structure of the SLX1–SLX4^SAP+CCD^ complex was determined by the single wavelength anomalous dispersion (SAD) method using endogenous Zinc ions as the anomalous scatterers. The 2.8 Å SAD data were collected at the wavelength of 1.2815 Å. The crystal belongs to the *P*2_1_2_1_2_1_ space group and there are two SLX1–SLX4 heterodimers per asymmetry unit. Four Zn ions were found using ShelxD (29), and the initial electron density map was generated by PHENIX (30). A model of the SLX1–SLX4^SAP+CCD^ complex was built and refined with a 2.5 Å dataset collected at 0.9792 Å using PHENIX and COOT (31).

The 1.5 Å SLX1–SLX4^CCD^ crystal diffraction dataset was collected at 0.9789 Å. The crystal belongs to the *P*2_1_2_1_2 space group, and there is one SLX1–SLX4 heterodimer per asymmetry unit. The structure was solved by molecular replacement using the MOLREP (32) program, with the 2.5 Å SLX1–SLX4^SAP+CCD^ structure as the search model. The 3.3 Å SLX1-SLX4^SAP+CCD^-5'-flap DNA complex data were also collected at 0.9789 Å, and the crystal belongs to the *P*6_5_22 space group with one SLX1–SLX4 heterodimer and one 5′-flap DNA per asymmetry unit. The structure was determined by molecular replacement using the 1.45 Å SLX1–SLX4^CCD^ structure as the search model. Further refinements using the 1.45 Å structure as the starting model produced electron density maps allowing unambiguous building of the model of SLX4 SAP domain and DNA, and the complete model of SLX1, SLX4 CCD and SAP domains and DNA was subjected to multiple rounds of refinement and structure adjustment. All structural refinements were carried out using PHENIX and the models were built and adjusted using COOT. Detailed statistics for crystallographic analyses can be found in Table 1.

**Table 1.** Data collection, phasing and refinement statistics

	SLX1–SLX4^SAP+CCD^ SAD	SLX1–SLX4^SAP+CCD^ Native	SLX1–SLX4^CCD^	SLX1–SLX4^SAP+CCD^-5'flap DNA
**Data collection**				
Space group	*P*2_1_2_1_2_1_	*P*2_1_2_1_2_1_	*P*2_1_2_1_2	*P*6_5_22
Cell dimensions				
*a*, *b*, *c* (Å)	61.01, 75.92, 186.83	61.28, 75.96, 186.97	70.30, 118.42, 62.00	123.22, 123.22, 233.58
*α*, *β*, *γ* (°)	90, 90, 90	90, 90, 90	90, 90, 90	90, 90, 120
Resolution (Å)	50.00–2.80(2.90–2.80)	50.00–2.50(2.59–2.50)	50.00–1.45(1.50–1.45)	50.00–3.30(3.42–3.30)
*R*_merge_	0.135(0.547)	0.104(0.383)	0.060(0.502)	0.122(0.722)
*I* / σ*I*	25.2(6.5)	15.7(3.9)	26.8(3.3)	20.1(3.7)
Completeness (%)	100.0(100.0)	99.3(98.3)	99.8(100.0)	99.8(100.0)
Total/Unique reflections	312 930/22 224	174 439/31 535	517 405/92 349	180 712/16 540
Redundancy	14.1(14.1)	5.5(5.6)	5.6(5.6)	10.9(11.1)
**Refinement**				
Resolution (Å)		50.00–2.50(2.59–2.50)	50.00–1.45(1.47–1.45)	50.00–3.30(3.50–3.30)
No. reflections		30829	92234	16509
*R*_work_/*R*_free_		0.201(0.264)/0.262(0.327)	0.123(0.161)/0.157(0.225)	0.235(0.311)/0.267(0.356)
No. atoms				
Protein		5945	3214	3352
DNA				1119
Ligand/ion		16	17	2
Water		125	698	12
*B*-factors (Å^2^)				
Protein		54.2	21.0	91.8
DNA				85.0
Ligand/ion		59.0	34.9	120.9
Water		47.3	39.2	59.6
R.m.s deviations				
Bond lengths (Å)		0.004	0.007	0.004
Bond angles (°)		0.740	1.036	0.993
Ramachandran plot				
Favored		687(98.1%)	365(99.2%)	389(97.7%)
Allowed		13(1.9%)	3(0.8%)	9(2.3%)
Outlier		0	0	0

*One crystal was used for each structure. *Values in parentheses are for highest-resolution shell.

### Single turnover nuclease assay

Each reaction mixture contains 4 pM Cy3-labeled DNA substrate added to a buffer containing 20 mM Tris, pH 8.0, 150 mM NaCl, 5% glycerol, 1 mM MgCl_2_ and 16 pM SLX1–SLX4 wildtype or mutant complex was incubated for 20–50 min at 37°C. The reaction was terminated by adding 2 mg/ml proteinase K for 15 min at 37°C. Reaction products were examined by native (1× TBE, 12%) or denatured (8 M urea, 20%) PAGE and analyzed by a fluorescence gel imaging system (BioRad GelDocEZ).

### Circular dichroism (CD) analysis

Far-UV CD spectra of wild-type and mutant SLX1–SLX4^SAP+CCD^ complexes were measured in the wavelength range of 200–250 nm on a Chirascan Plus CD instrument (Applied Photophysics, UK) at 25°C in a 1 mm path-length thermostated cuvette, with the protein samples at 0.2 mg/ml in 20 mM Tris, pH 8.0, and 200 mM NaCl. Data were collected with a band pass of 1 nm and the sensitivity was set to 100 mdeg.

### Fluorescence polarization assay (FPA)

FPA was performed according to a published protocol (33) with minor modifications. Custom-synthesized 5′-FAM-labeled Flap-15nt DNA (Takara) was mixed at 100 nM with increasing amounts of SLX1–SLX4 complex in a buffer containing 20 mM Tris, pH 8.0 and 100 mM NaCl. The mixtures were incubated for 30 min at room temperature. The measurements were performed on an Envision multimode plate reader (PerkinElmer). The background mP values (no protein) were subtracted and the *K*_D_ values were calculated by nonlinear regression fitting of specific binding with Hill slope model for the SLX1–SLX4 complex and using the GraphPad Prism 8 software. Saturation levels were calculated as [saturation] = [mP (measured) − mP (background)]/mPmax (calculated).

### Molecular dynamics (MD) simulation

Two systems, named Group 1 and Group 2, were subjected to MD simulations. The starting models for both simulations were derived from the *Sc*SLX1–SLX4^SAP+CCD^-5'-flap DNA crystal structure, except that in Group 2 a 10-nt nucleotide flap (5′-TGCCTTGCTA-3′) substitutes the 1-nt flap DNA in the crystal structure and the 5′ tail is manually placed at an arbitrary orientation not contacting SLX. For each system, missing residues and hydrogen atoms were added using SWISS-MODEL (34). The protonation states of histidine residues were assigned as predicted by H++ (35). Zinc ions observed in the crystal structure were retained and the chelating cysteine and histidine residues were deprotonated. The Amber FF14SB (36) and Parmbsc1 (37) force fields were used for protein and DNA, respectively. The complex was solvated using the TIP3P model in a hexagonal explicit water box under the periodic boundary condition, and a distance of 12 Å between box edges and the closet atoms of the complex is imposed. Na^+^ was added as counter ions to neutralize each system.

For each solvated system, a 5000-step energy minimization for the whole residues was performed, followed by a combined equilibration process with a 500-ps constant volume ensemble to heat the system from 0 to 300 K, and a 500-ps constant pressure ensemble at a constant pressure of 1 bar. During equilibration, a force constant of 10 kcal·mol^–1^·Å^–2^ as a harmonic constraint was applied. Then, 1-μs MD simulation of each system was performed using the AMBER18 software package in constant pressure ensembles at 300 K with the constraint released. The time step was set to 2 fs, and the SHAKE algorithm was used to restrain all of the bond lengths involving hydrogen atoms. The particle Mesh Ewald (PME) method was used to calculate the long-range electrostatic contributions. The cut-off value of the van der Waals interactions was set to 10 Å.

## RESULTS

### Overall structure of *S. cerevisiae* SLX1–SLX4 complex

We first assembled the complex of full-length yeast SLX1 and a C-terminal fragment of SLX4 encompassing the SAP and CCD domains (SLX4^SAP+CCD^, a.a. 610–748) by co-expression in *E. coli* (Figure 1A), then crystallized and solved a 2.5 Å structure. However, the SAP domain of SLX4 is disordered, and there are two SLX1–SLX4 heterodimers per asymmetric unit (ASU), which occurs through intermolecular contact via the CCD domains of SLX4 and is likely a crystallization effect (Supplementary Figure S1). Since the SAP domain is completely missing in the structure, we then expressed only the SLX4^CCD^ domain (a.a. 675–748) together with SLX1 and solved a 1.45 Å structure by molecular replacement (Figure 1B, Table 1). In the new crystal form, there is one SLX1–SLX4^CCD^ heterodimer per asymmetric unit. Superposition of the SLX1–SLX4^CCD^ and the SLX1–SLX4^SAP+CCD^ structures shows that the two heterodimers are highly similar, except that the loop connecting α1 and β3, and the one between α2 and α3 in the Uri domain appear to have variable conformation (Supplementary Figure S2). With this caveat in mind, we shall use the higher resolution SLX1–SLX4^CCD^ structure for analysis of the apo SLX1–SLX4 complex.

In the *Sc*SLX1–SLX4^CCD^ structure, all but the very N-terminal six residues of SLX1 are well defined (Figure 1B). The Uri domain in SLX1 adopts an α/β sandwich configuration common to the GIY-YIG nuclease superfamily (Supplementary Figure S3). Two zinc ions are bound in the Zinc-finger (ZF) domain of SLX1, which is formed by two α-helices and four short β-strands. Both the Uri and ZF domains, which are connected by a long α-helix, interact with the CCD domain of SLX4. The globular CCD domain is composed of five α-helices, among which, α2 and α5 contact the ZF and Uri domains of SLX1, respectively (Figure 1B). SLX4^CCD^ and SLX1^ZF^ interaction buries 556 Å^2^ surface area and occurs mainly via hydrophobic residues (Figure 1C). Aromatic sidechains of Tyr703 and Phe705 of the CCD domain contact either aromatic or hydrophobic sidechains of His256, Trp288, Ile273 and Val292 of the ZF domain. In comparison, the 520 Å^2^ interface between SLX4^CCD^ and SLX1^Uri^ shows mainly polar interactions (Figure 1D). Notably, Asp737 of CCD makes a hydrogen bond with His92 of SLX1^Uri^, and mainchain carbonyl groups of Leu743 and Val738 of CCD form hydrogen bonds with the sidechains of Gln80 and Arg128 of Uri, respectively. The packing of SLX1 Arg128 against the C-terminal end of CCD helix α5 via charge-helix dipole interaction, as well as indirect contact via ordered water molecules, additionally stabilized the packing between the Uri domain of SLX1 and the CCD domain of SLX4.

**Figure 2.**
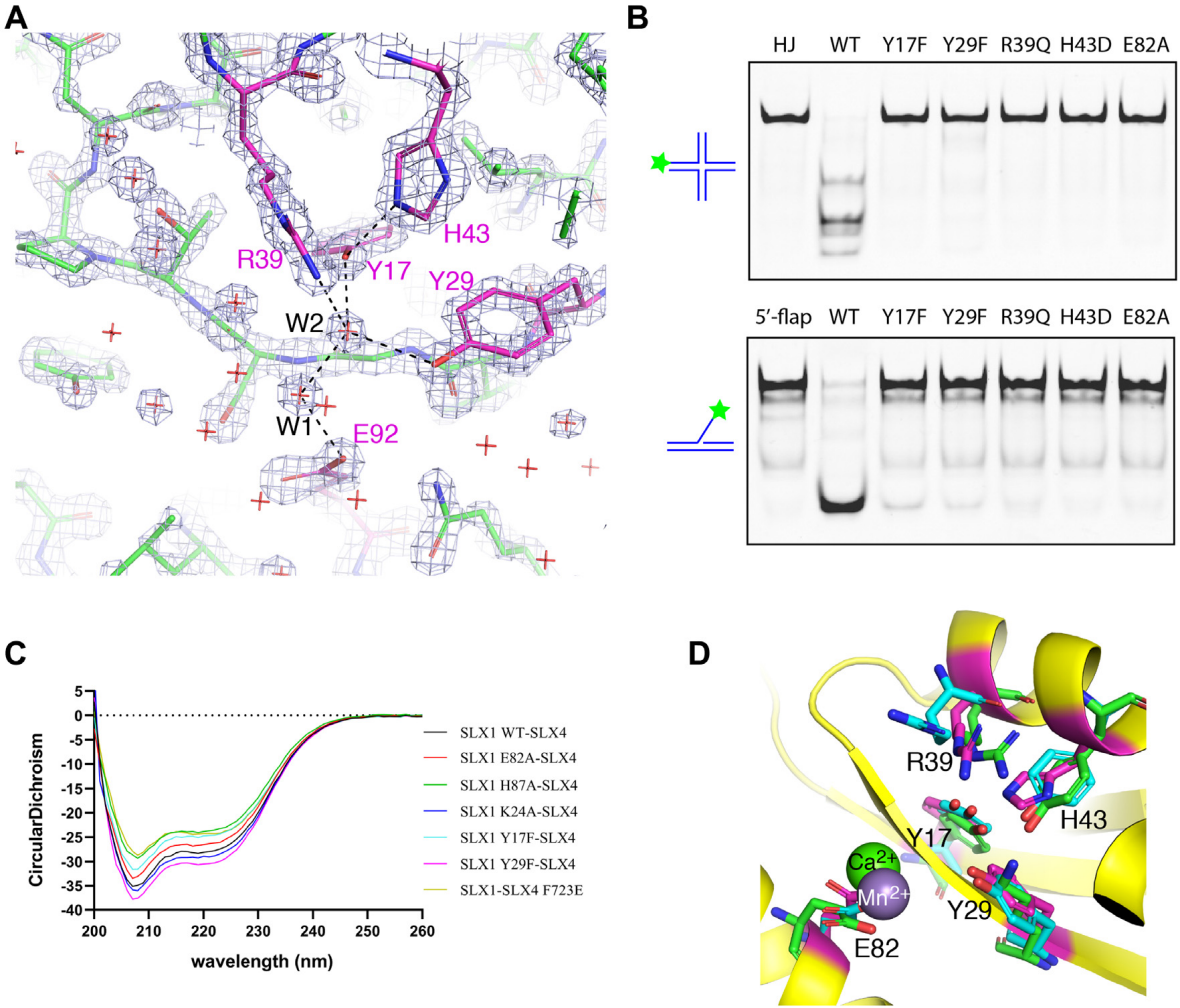
The catalytic active site of SLX1. (**A**) A section of the 2*F*_o_ – *F*_c_ electron density map, contoured at 3.5 σ, covering the active site. A stick model of the refined structure is superimposed. The carbon half-bonds of the active site residue are labeled magenta, and the rest is shown in green, while nitrogen and oxygen half-bonds are shown in blue and red, respectively. Red crosses indicate water molecules, and two of which making hydrogen bonds to catalytic residues are designated W1 and W2. (**B**) Mutation of SLX1 active site residues results in impaired cleavage of HJ (top panel) and 5′-flap DNA (bottom panel) substrates. The assay was carried out using the protein complexes assembled from wild-type (WT) or indicated SLX1 mutants assembled with SLX4^SAP+CCD^, and a 5′-Cy3 labeled HJ and 5′-flap DNA as the substrates (Supplementary Table S1). (**C**) Circular dichroism (CD) spectra of WT and mutant SLX1–SLX4^SAP+CCD^ complexes show that protein folding was not compromised by mutations. (**D**) Superposition of Hpy188I (green; PDB ID: 3OR3) and UvrC (cyan; PDB ID: 1YD0) GIY-YIG domains with the Uri/GIY-YIG domain of SLX1 shows that the active sites are highly conserved. A calcium ion from the Hpy188I structure (green sphere), and a manganese ion (grey sphere) in the UvrC structure offer insights into the role of the conserved glutamate residue.

The overall structure of the *Sc*SLX1–SLX4^CCD^ complex is very similar to that of its *C. glabrata* and *T. terrestris* counterparts (Supplementary Figure S4). The catalytic active site of *Sc*SLX1 is formed by five conserved residues including Tyr17, Tyr29, Arg39, His43 and Glu82 located in the Uri domain (Figure 2A, Supplementary Figure S3), and they are spatially well aligned among the three structures (Supplementary Figure S4). These five residues are essential for DNA cleavage, as demonstrated by dramatic loss of SLX1’s nuclease activity with individual amino acid substitution, whether using a HJ or a 5′-flap DNA as the substrate (Figure 2B). The loss of nuclease activity of these mutants is not due to protein misfolding as a result of amino acid substitution, as judged by circular dichroism analyses (Figure 2C). Tyr17, Arg39 and Glu82 are invariant within the GIY-YIG superfamily, and Tyr29 is highly conserved but a rare exception with a lysine is found in Hpy188I (38), while His43 is more readily substituted by a tyrosine (39) (Figure 2D). The invariant glutamate residue has been shown to bind a metal ion, while the rest of the catalytic residues are implicated in direct DNA binding or coordinating water molecules for catalysis (Figure 2D). Our structure does not have a metal ion bound to Glu82, but a pair of well-ordered water molecules form a network of hydrogen bonds connecting the active site residues (Figure 2A). The two ordered water molecules occupy conserved positions in the UvrC structure, suggesting a shared catalytic mechanism of GIY-YIG nucleases (40).

### Biochemical function of the SAP domain of SLX4

**Figure 3.**
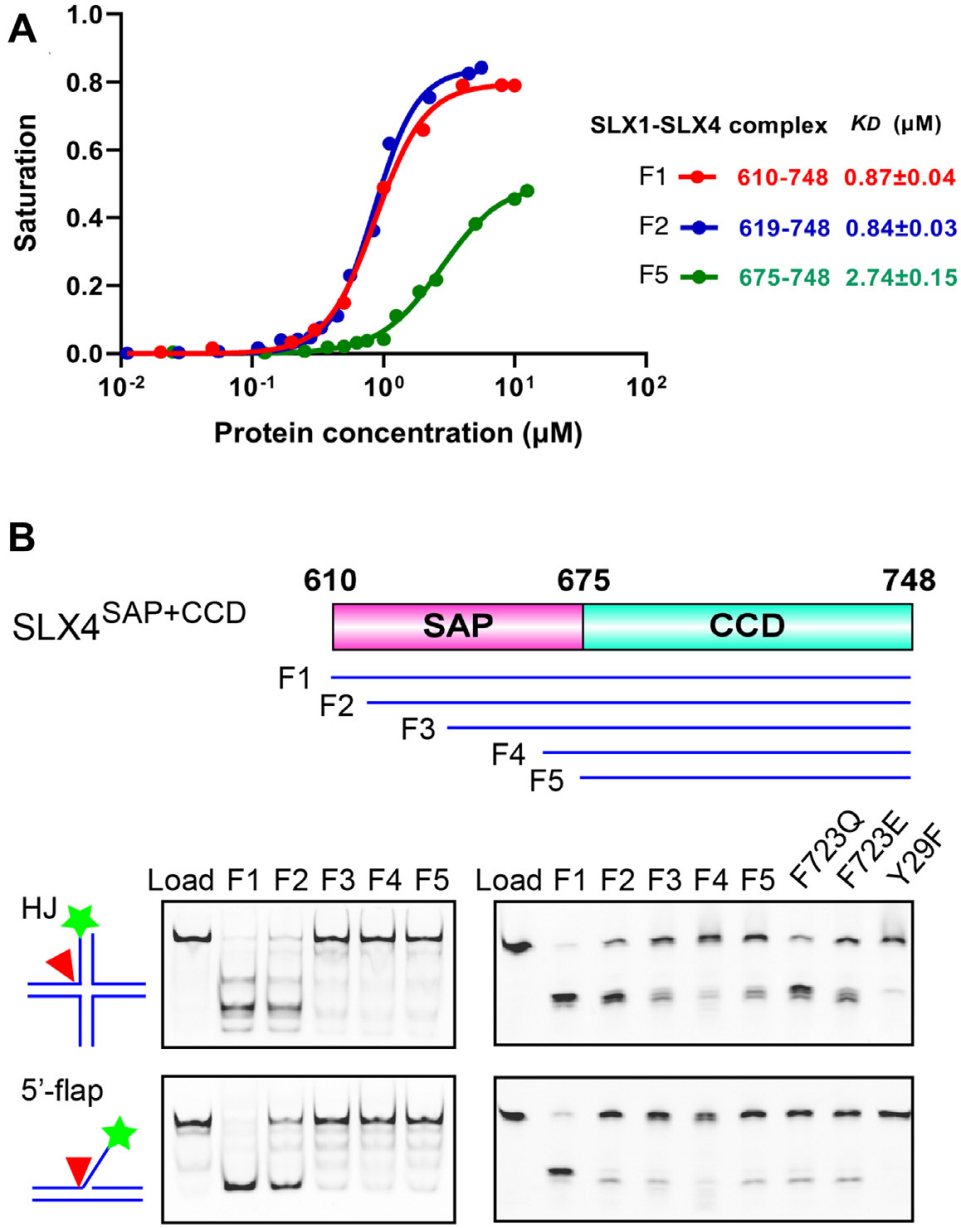
Biochemical functions of the SAP domain of SLX4. (**A**) The SAP domain of SLX4 enhances DNA binding of the SLX1–SLX4 complex. Fluorescence Polarization Assay (FPA) assessment of the binding of SLX1–SLX4 complexes with the indicated SLX4 fragments to 5′-flap DNA. The derived *K*_D_ values are displayed. (**B**) The SAP domain of SLX4 is required for efficient and accurate cleavage of DNA substrates. Top panel, schematics of truncation fragments of SLX4^SAP+CCD^ used for assessing the function of SAP domain; middle and bottom left panels, cleavage of HJ and 5′-flap DNA by complexes formed between SLX1 and the indicated SLX4 fragments, assessed by native gel electrophoresis, respectively; middle and bottom right panels, analysis of the indicated SLX4 mutant complexes by denatured gel electrophoresis, respectively. The SLX1 Y29F mutant complex with the F1 fragment of SLX4 is shown as a negative control.

All SLX1–SLX4 structures determined so far, including ours, did not provide structural and functional insights into the SAP domain of SLX4, despite being included in our crystallization specimen. Based on its predicted biochemical function, we reasoned that it might not be stabilized in the absence of DNA (19,20). To reveal its role in DNA binding and impact on DNA cleavage activity, we tested these properties using SLX4 fragments with or without the SAP domain. Our electrophoretic mobility shift assay (EMSA) shows that SLX4^SAP+CCD^ shifted both HJ and 5′-flap DNA, while the shorter SLX4^CCD^ fragment showed no detectable binding to either type of DNA (Supplementary Figure S5A). An analysis by fluorescence polarization assay (FPA) reveals that the SAP+CCD fragment binds 5′-flap DNA ∼25 times stronger than the CCD fragment does, with *K*_D_ values of 1.4 and 34.2 μM, respectively (Supplementary Figure S5B). These results indicate that the SAP domain of SLX4 is endowed with an intrinsic DNA binding property. Consistently, upon forming a complex with SLX1, the SLX4^SAP+CCD^ complex binds 5′-flap DNA with a *K*_D_ value of 0.9 μM, which is approximately 3 times stronger than the one with SLX4^CCD^ (Figure 3A).

To test the nuclease activity of the SLX1–SLX4 complexes assembled from SLX4 fragments with successive truncations into the SAP domain, a DNA strand labeled with Cy3 at its 5′ end is used to form HJ or 5′-flap DNA substrates for analysis of the enzymatic activity of these complexes (Figure 3B). Compared with the intact SLX1–SLX4^SAP+CCD^ complex (F1: a.a. 610–748), deletion of merely nine residues into the SAP domain (F2: a.a. 619–748) already results in an appreciable level of reduction of the cleavage activity, especially with the 5′-flap DNA substrate, although the 5′-flap DNA binding abilities are comparable for the two complexes (Figure 3A). Further truncations (F3–F5) into the SAP domain result in severer loss of the nuclease activity. Evidently, the reduced DNA cleavage activity is also accompanied with cuts at alternative and/or non-specific DNA sites, as judged by the nature of the product bands revealed in the denaturing gel (Figure 3B). The above experiments demonstrate that the SAP domain of SLX4 is not only crucial for DNA binding, it is also important for DNA cleavage activity and cleavage site selection of the SLX1–SLX4 complex.

### Structure of SLX1–SLX4^SAP+CCD^ in complex with 5′-flap DNA

**Figure 4.**
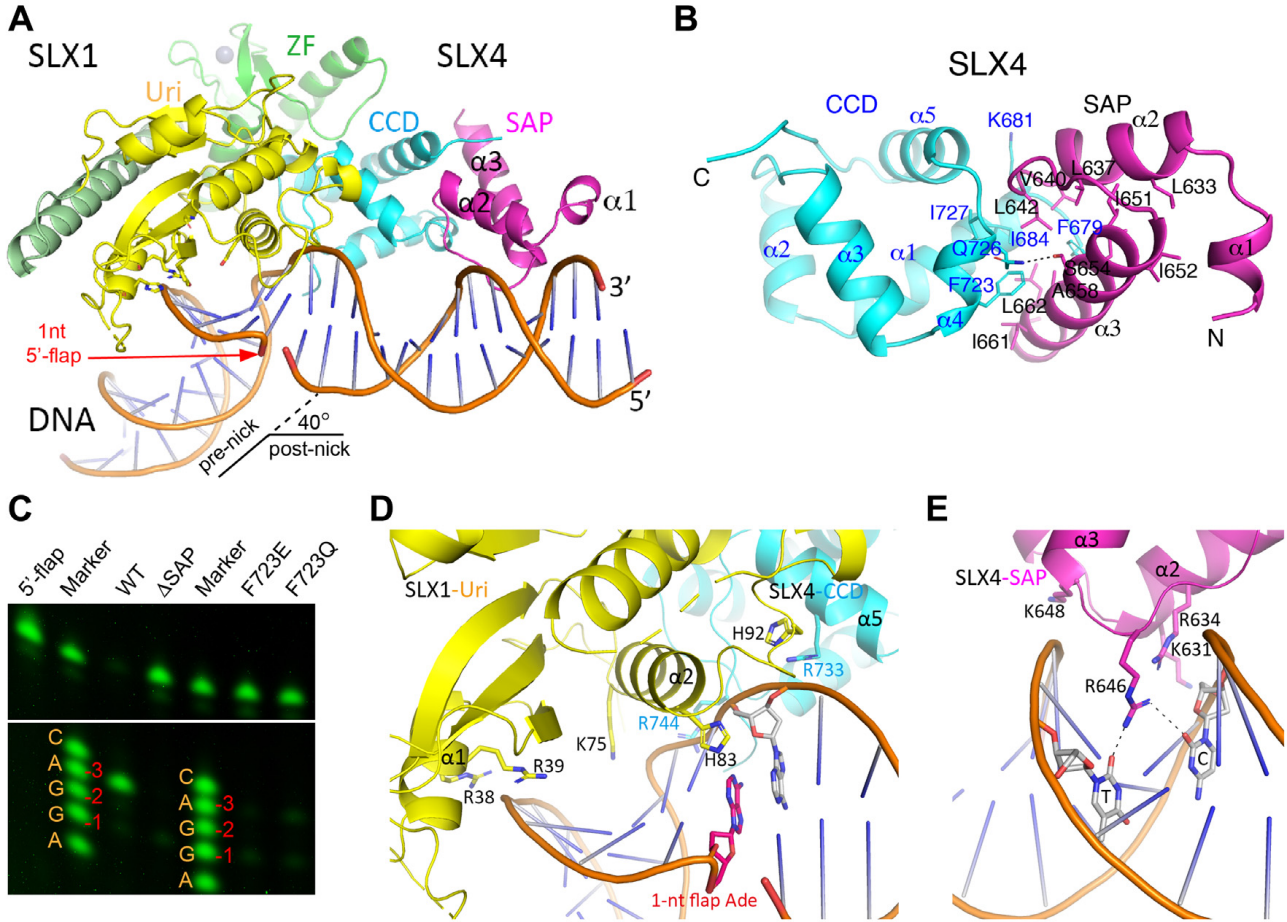
Structure of SLX1–SLX4^SAP+CCD^ bound to a 1-nt 5′-flap DNA. (**A**) A cartoon representation of the structure, with the SAP domain of SLX4 colored in magenta, DNA in orange, and the rest of the protein domains as in Figure 1. The location of the 1-nt flap is indicated with a red arrowhead. (**B**) A detailed view of packing interaction between the CCD and SAP domains of SLX4. Note that the conserved F723 of the CCD domain occupies a central position at the inter-domain interface. (**C**) Interference of the CCD-SAP interaction by the F723E and F723Q mutants impairs cleavage at the -3 position exhibited by the WT SLX1- SLX4^SAP+CCD^ complex, much like with the removal of the entire SAP domain (ΔSAP), as judged by single-nucleotide resolution gel electrophoresis. The DNA marker is a mixture of synthetic DNA oligos all with a 5′-Cy3 label, carrying the XO-1 oligo sequence, as listed in Supplementary Table S1 (also see the right bottom panel of Figure 5D), but with the 3′-most nucleotide ending at –4 (C), –3 (A), –2 (G), –1 (G), +1 (A), etc., as labeled. (**D**) Protein-DNA interaction involving the Uri domain of SLX1 (yellow) and the CCD domain of SLX4 (cyan). The involved residues are shown as sticks. (**E**) The SAP domain of SLX4 (magenta) contacts DNA principally via charge interaction, and the involved residues and bases are shown.

To understand how the SAP domain achieves its function, we set out to obtain a SLX1–SLX4^SAP+CCD^ structure in complex with DNA. After screening a large variety of DNAs, we succeeded in crystallizing SLX1–SLX4^SAP+CCD^ with a 26-bp dsDNA containing a 1-nt 5′-flap in the middle and solved a 3.3-Å structure (Figure 4A and Supplementary Figure S6A). All of the residues and nucleotides are well defined in the structure except two loop regions, one connecting the SAP and CCD domains of SLX4 (a.a. 667–677), and the other segment linking α1 and β2 near the active site in SLX1^Uri^ (a.a. 49–55) (Supplementary Figure S6B).

The overall structure of SLX1, SLX4 and their interface remain mostly the same as in the apo structure of SLX1–SLX4^SAP+CCD^, except that the SAP domain of SLX4 is now visible (Figure 4A). Two α helices, α2 and α3, form the core of the SAP domain, and amino acid residues located at their N-terminal portions and the loop connecting the two helices bind the minor groove of DNA approximately one turn away from the flap junction, in the direction termed the post-nick side (3′ side of the uncut strand). The two SAP helices pack together principally via hydrophobic interactions (Figure 4B). The SAP domain packs against the CCD domain via its C-terminal portion and the α1 and α4 helices of CCD, although the linker connecting the two domains is disordered (Supplementary Figure S3B). The mainly hydrophobic interdomain interactions involve Val640, Leu642, Ala658, Ile661 and Leu662 of the SAP domain, and Phe679, Ile684, Phe723 and Ile727 of CCD (Figure 4B). It is interesting that Phe679 and Phe723 occupy central positions at the interface of the two SLX4 domains in the presence of DNA, as they were seen mediating the formation of a non-physiological dimer of the SLX1–SLX4^SAP+CCD^ complex within the crystal asymmetric unit (Supplementary Figure S1A and S1B). This observation suggests that, in the absence of DNA, the SAP domain is not stably positioned against the CCD domain, thus exposing the two phenylalanine residues on the CCD domain for engagement. Finally, a hydrogen bond between the hydroxyl group of Ser654 in the SAP domain and the sidechain amino group of CCD’s Gln726, and packing of Lys681 from the CCD domain against the C-terminal end of SAP’s α2 helix also contributes to the juxtaposition of the two domains (Figure 4B).

Disruption of the interdomain interaction by mutating Phe723 of CCD either to a glutamine or glutamate results in a much less active SLX1–SLX4^SAP+CCD^ complex (Figure 3B, bottom right panels). The nuclease activities of the Phe723 mutant complexes are similar to that of the SLX1–SLX4^CCD^ complex without the SAP domain (F5 fragment). In the denaturing gel, the major cleavage products of 5′-flap DNA by the Phe723 mutant or the SAP-deleted SLX1–SLX4^CCD^ complexes appear to be shorter than that generated by the wild type enzyme complex (Figure 3B). High resolution gel electrophoresis reveals that the wild-type enzyme complex cuts 5′-flap DNA at the -3 phosphodiester bond (between –3 and –4 nucleotides) much more frequently than at the –2 and –1 positions, while deletion of the SAP domain greatly reduced the nuclease activity and shifts the major cleavage site to the -1 position (Figure 4C). The F723Q or F723E mutant of SLX4 also results in much less active enzyme complexes and the cleavages occur at both the –3 and –1 positions. This debilitating effect of the Phe723 mutations are not due to protein misfolding (Figure 2C). These results clearly demonstrated the important role of the SAP domain in substrate recognition and cleavage site selection of the SLX1–SLX4 complex.

SLX1–SLX4^SAP+CCD^ interacts with the 5′-flap DNA through positively charged regions of the Uri domain of SLX1, the C-terminal part of CCD and the SAP DNA binding unit of SLX4 (Supplementary Figure S6C). Besides the catalytic core, several positively charged residues of SLX1^Uri^, including Arg38 and Arg39 from α1, Lys75 and His92 from α2 and the following loop, contact the backbone phosphate groups of DNA (Figure 4D). Similarly, the DNA backbone also interact with Arg733 and Arg744 of SLX4^CCD^, and Lys631, Arg634 and Lys648 of SLX4^SAP^ via charge interactions (Figure 4D and E). Arg646 of SLX4^SAP^ inserts into the minor groove of the post-nick part of DNA and forms hydrogen bonds with the carbonyl groups of the pyrimidine rings of a Cyt and a Thy. Surprisingly, the 1-nt flap, an adenine, does not project away from the DNA duplex, instead, its base intrudes into the duplex and stacks with adjacent bases and contacts the imidazole ring of His83 of SLX1^Uri^ (Figure 4D). The insertion of the extra base distorts the local double helix structure and allows the bending of DNA by approximately 40 degree (Figure 4A). The most significant distortion is observed with the continuous (non-cleaved) strand, where the intrusion of the 1-nt flap from the opposite strand makes two neighboring bases on each side of the wedge ∼7 Å apart, instead of the ∼3.4 Å in canonical B DNA, and at least four consecutive nucleotides on the continuous strand next to the wedge adopting a C3′-endo sugar pucker.

### DNA binding mode of the SLX1–SLX4 complex

**Figure 5.**
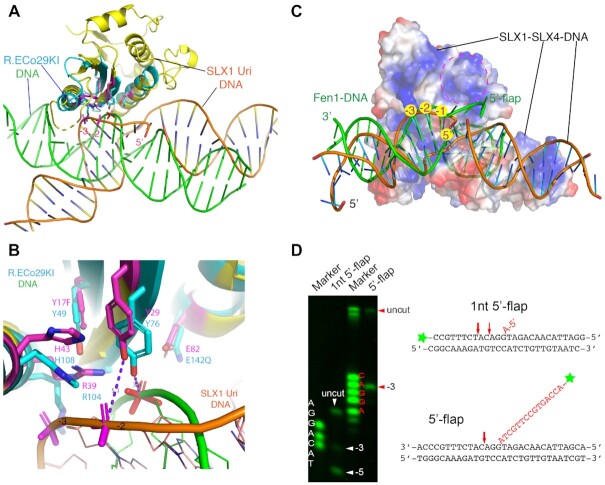
Comparison of DNA binding modes of nucleases. (**A**) Superposition of the SLX1–SLX4^SAP+CCD^-DNA complex structure (yellow) with that of the R. Eco29KI-DNA complex (PDB: 3MX4, cyan) shows the difference of DNA conformation and positioning of the DNA cleavage sites. The structures were aligned via their Uri nuclease domains, and SLX4 is not shown for viewing clarity. The DNA bound to R. Eco29kI is colored green, and that of SLX1 is shown in orange. Active site residues in both enzymes, and nearby DNA backbone phosphate groups are shown in a stick model, colored magenta for the SLX1 complex, and cyan and red for that of the R. Eco29KI complex. (**B**) A close-up view of the superimposed active sites. (**C**) Superposition of the 5′-flap DNA substrate of FEN1 (green; PDB ID: 5KSE) with the 1-nt 5′-flap DNA of the SLX1–SLX4 complex, which is shown in a semi-transparent electrostatic potential surface representation. Three DNA basepairs next to the flap junction (–1 to –3 position) were used for alignment. The superposition shows notable differences of DNA structure at the pre-nick end of DNA, and the post-nick portion of FEN1 DNA is nearly perpendicular to the SLX1–SLX4 DNA. The 5′-flap of FEN1 DNA is next to an open surface area, indicated by a dashed-line circle, enriched with positively charged residues. (**D**) Sequencing gel (left panel) shows that SLX1–SLX4 cuts 1-nt 5′-flap DNA mainly at the –5 position, in contrast to the –3 position with DNA having a longer 5′-flap. The DNA marker for the 1-nt 5′-flap DNA substrate is a mixture of synthetic oligos of difference length, all with a 3′-Cy3 label, starting at the –2 nucleotide following the sequence of the flap strand displayed in the right panel. The marker for the 5′-flap DNA substrates is the same as that used in Figure 4C, as also displayed in the right panel.

Together with the *Tt*SLX1–SLX4^CCD^–DNA structure, the available structural information still appears insufficient to account for the DNA binding mode of the SLX1–SLX4 complex capable of productive DNA cleavage. In the *Tt*SLX1–SLX4^CCD^–DNA complex, the single-stranded stem-loop DNA is bound at a positively charged surface region on the side of the SLX1^Uri^ domain separated from the catalytic pocket, therefore not accessible for DNA cleavage (Supplementary Figure S7). In our structure, the DNA region near the active site is distorted from the canonical double-stranded form and out of immediate reach by the catalytic residues (Figure 5A and B). Alignment of our SLX1–SLX4–DNA complex structure with the structure of the prototypical type-II GIY-YIG nuclease-DNA complex, the R. Eco29KI-DNA complex (41), shows that the two active sites, which are made up of five identical residues, are spatially conserved (Figure 5A and B). Though the nearest phosphodiester bond of the 5′-flap DNA to the active site of *Sc*SLX1–SLX4^SAP+CCD^ is between the –2 and –3 position, which is only a minor cleavage site on the 5′-flap DNA (Figure 4C). The structures show that the distances between the hydroxyl group of a catalytic tyrosine (Y29 in *Sc*SLX1) to the nearest backbone phosphorus atom are 3.6 Å in the R. Eco29KI–DNA complex and 5.1 Å in the *Sc*SLX1–SLX4^SAP+CCD^–flap DNA complex (Figure 5B). The former arrangement more readily accounts for a catalytically productive DNA binding.

Possible causes for the non-ideal DNA binding in the *Sc*SLX1–SLX4 ^SAP+CCD^-DNA structure may be twofold. First, insertion of the one 5′-flap base into the duplex distorted the local structure of DNA (Figure 4A). Second, the DNA is bent by ∼40 degree around the duplex-flap junction, compared with the continuous DNA substrate of Eco29KI-DNA, which is a restriction endonuclease (Figure 5A). These differences in the DNA structure have two consequences. One is that helix α1 and the following loop in SLX1 are not engaged in binding the major groove of DNA in the pre-nicked portion, as in the case of the Eco29KI-DNA structure. The other is that the –2 phosphodiester bond, instead of the major –3 cleavage site, is placed closest to the active site (Figure 5A and B).

To gain better insights into productive 5′-flap DNA bindings, we turned to the DNA binding mode of the well-studied Flap Endonuclease 1 (FEN1), which plays important roles in DNA replication and repair (42,43). Due to lack of structural similarity between the proteins, we aligned the –1 to –3 paired region in the pre-nicked portion of DNA (Figure 5C and Supplementary Figure S8). The comparison shows that the minor groove in the pre-nicked end of DNA in the SLX1–SLX4 complex is significantly narrowed, and the backbone of the continuous strand is kinked at the position facing the –3 nucleotide on the incised strand (Figure 5C). The post-nicked portion of DNA in the FEN1 structure lies almost perpendicular to the pre-nicked region, as well as with respect to the post-nicked region of DNA in the SLX1–SLX4 structure (Supplementary Figure S8). The 5′-flap of FEN1 substrate DNA exits a gateway capped by an α-helix, while the projected binding site for 5′-flap in SLX1 is an open, positively charged surface area (Figure 5C and Supplementary Figure S8).

### Recognition of the 5′-flap DNA structure

In our nuclease activity assay, two cleavage product bands could be detected when the 1-nt 5′-flap DNA was used as a substrate. The minor band corresponds to cleavage at the –3 position, while the major band actually corresponds to cut at the –5 position (Figure 5D). The intensities of the cut and uncut bands indicate that 1-nt 5′-flap DNA is not a very good substrate compared to the longer, 15-nt 5′-flap DNA substrate, where the cleavage predominantly occurs at the –3 position (Figure 5D). Thus, recognition of the longer 5′-flap has a significant impact on the enzymatic property of the SLX complex toward 5′-flap DNA substrates. For the 1-nt 5′-flap substrate, the crystal structure appears to represent an inhibitory conformation, as the scissile phosphate is out of reach by the catalytic residues (Figure 5B). Two reasons could possibly account for this setting: first, wedging of the 1-nt 5′-flap nucleotide into the duplex may prevent correct positioning of the scissile phosphate next to the catalytic residues; second, crystal packing conceivably stabilizes the pre-nicked portion of DNA in an unproductive configuration (Supplementary Figure S9). Hence, an understanding of the manner by which 5′-flap DNA binds the SLX1–SLX4 complex in a productive state is needed to comprehend the molecular details of the catalytic process. Unfortunately, we were not able to crystallize the *Sc*SLX1–SLX4^SAP+CCD^ heterodimer in complex with DNA having longer 5′-flaps, despite many efforts.

**Figure 6.**
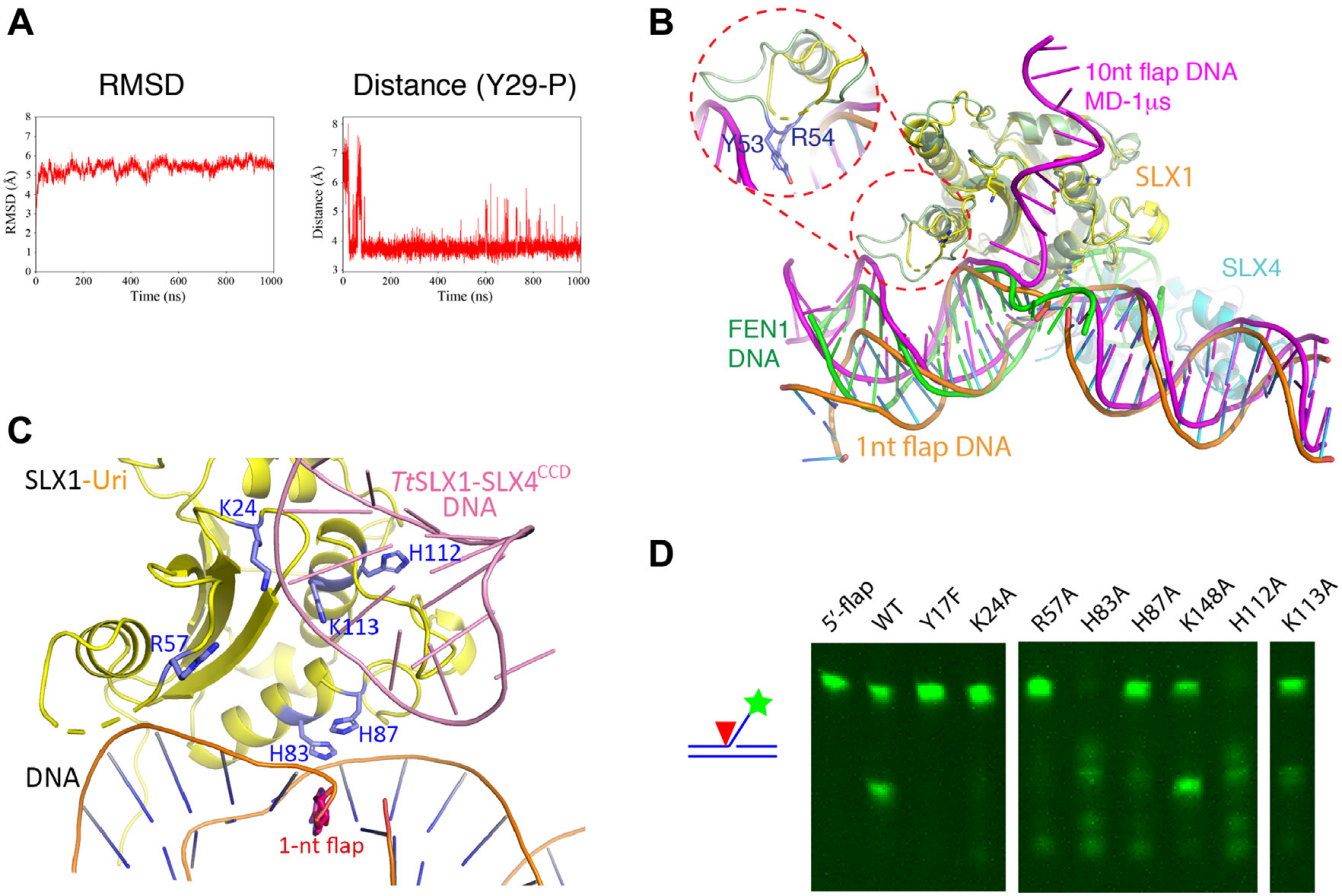
Recognition of the 5′-flap by the SLX1–SLX4 complex. (**A**) MD simulation of a 10-nt 5′-flap DNA bound to the SLX1–SLX4^SAP+CCD^ complex. The RMSD values and the distances between the catalytic Tyr29 and the neighboring DNA backbone phosphate during the course of 1 μs simulation are shown. (**B**) A representative model from the MD simulation (SLX1 in pale green, SLX4 in light blue, and DNA in magenta) shows that the 5′-flap binds SLX1 in a shallow cleft flanked by positively charged residues (also see Supplementary Figure S10). The crystal structure of the SLX1–SLX4^SAP+CCD^–DNA complex (SLX1 in yellow, SLX4 in cyan and DNA in orange) and the 5′-flap DNA substrate of FEN1 (green) are superimposed. The conformation of the pre-nick DNA from the MD simulation appears more similar to that of FEN1 DNA. The inset shows that the loop connecting α1 and β3 is capable of binding DNA, as demonstrated by the indicated residues in contacting the major groove of DNA. (**C**) Superposition of the hairpin DNA from the *Tt*SLX1–SLX4^CCD^–DNA complex (pink; PDB ID: 6SEI) onto the *Sc*SLX1–SLX4^SAP+CCD^–DNA structure. Selected positively charged residues are shown in a stick representation. For visual clarity, SLX4 is not displayed. (**D**) Mutations of selected residues in the newly identified DNA binding region of SLX1^Uri^ differentially affect the 5′-flap cleavage activity of the SLX1–SLX4^SAP+CCD^ complex.

To gain some initial insights, we evaluated the binding of the SLX1–SLX4 complex to DNA with a longer 5′-flap by molecular dynamics (MD) simulation using DNA with a 10-nt 5′-flap. The initial model was constructed using our *Sc*SLX1–SLX4^SAP+CCD^–DNA structure as the framework, and a 10-nt flap was placed at an arbitrary orientation not interacting with SLX1 at all. A 1-μs MD simulation shows that the protein-DNA complex is stabilized quickly in ∼30 ns, and the distance between the active site of SLX1 (the hydroxyl group of Tyr29) and the -2 phosphate group of DNA mostly stays at ∼3.7 Å after ∼80 ns of the MD procedure (Figure 6A). The stabilized model shows that the SLX1–SLX4 structure stays quite similar to the crystal structure throughout the MD simulation (Figure 6B). However, it is worth noting that, in the Uri domain of SLX1, the loop connecting α1 and β3 (a.a. 46–57), which is mostly disordered in the crystal structures, engages the major groove of DNA in the MD structure, notably via Tyr53 and Arg54, resembling the role of the corresponding Uri domain helix in the Eco29KI–DNA structure (Figures 5A and 6B). The DNA shows more prominent changes in the MD structure. First, the pre-nicked portion of dsDNA is raised toward SLX1 and the duplex is less distorted (Figure 6B). Second, the post-nicked portion extended further away from the flap junction. Finally, the freely placed 10-nt 5′-flap rests on the positively charged surface area overlapping with the DNA hairpin binding region in the *Tt*SLX1–SLX4^CCD^–DNA structure (Figure 6B, C and Supplementary Figure S10).

The MD simulation clearly shows the elasticity of the 5′-flap DNA substrate bound to the SLX1–SLX4 complex. Although the scissile (–3) phosphate group still appears one register away from the position for cleavage in the MD model, the –2 phosphate moved closer to the hydroxyl group of Tyr29 than in the crystal structure. It is not unprecedented that the scissile bond on nucleic acids will shift toward the catalytic active site in the presence of metal ions (43–45). On the other hand, the predicted 5′-flap-binding positively charged surface region can be readily verified. Mutation of selective positively charged residues in the region, including Lys24, Arg57, His83, His87, His112 and Lys113 in *Sc*SLX1, differentially impacts the nuclease activity of *Sc*SLX1–SLX4^SAP+CCD^ (Figure 6C and D). K24A, R57A, H87A and K113A mutants are less active, while H83A and H112A become more active than the wild type enzyme. All of these mutants cleave DNA non-specifically. These results confirm the importance of this positively charged region for the specificity and activity of the SLX1–SLX4 enzyme, and support our MD simulation result that this positively charged surface area accommodates the binding the single-stranded arm of a longer 5′-flap.

## DISCUSSION

In this study, we attempted to better understand the DNA binding mode and the mechanism governing DNA cleavage site selection of the SLX1–SLX4 complex. Given its roles in processing diverse forms of DNA substrates, we imagine that there might be multiple forms of DNA binding when the enzyme complex engages distinct types of DNA substrate. Here, we focused on dissecting these mechanisms on the 5′-flap DNA in this study. One of our important findings is that the SAP domain of SLX4 is critically important for the efficiency and accuracy of the enzymatic activity of the SLX1–SLX4 complex. None of the previous structures of the SLX1–SLX4 complexes contain the SAP domain, either because it was disordered or left out in the study. Our structure shows that it independently folds into a helical module, consisting of two long α-helices and a shorter, N-terminal helix. The SAP domain binds the minor groove of DNA approximately one turn away from the flap-duplex junction, principally via several positively charged residues. The importance of the SAP domain for DNA binding of SLX4 is confirmed by our *in vitro* binding experiments (Supplementary Figure S5). Interestingly, the SAP domain is stably placed against the CCD domain in the presence of DNA, in contrast to being flexibly tethered in the absence of DNA. Disturbance of the interdomain interaction by mutations in the CCD domain not only weakens the SLX1–SLX4 nuclease activity, it also results in cuts at alternative sites much like in the absence the SAP domain (Figures 3B and 4C).

The tandem SAP-CCD domain arrangement of SLX4 is evolutionarily conserved in eukaryotes. While the CCD domain is responsible for interaction with SLX1, there is no consensus about the function of the SAP domain. In human SLX4, it was reported that the SAP domain is not required for processing of HJ and removal of 5′-flap from a splayed-arm structure (7). It is perhaps easier to understand that the SAP domain may be dispensable in processing 5′-flap in a splayed-arm DNA structure, as there is no immediate double-stranded region in the post-nicked region of DNA. The reason is not clear in the case of HJ resolution. Nevertheless, several possibilities may account for the difference. First, the SAP domain may have a different role in HJ resolution and removal of the single-stranded arm of a 5′-flap DNA. Increasing evidences indicate that the SLX1–SLX4 heterodimer forms a larger complex with the MUS81–EME1 complex via the SLX4 scaffold in resolution of HJs: the SLX1–SLX4 complex will make an initial cut to generate a nicked HJ intermediate, which then activate the MUS81–EME1 complex to cut at a site across the junction, leading to the eventual resolution of the HJ into linear DNA products (6,21). The SAP domain of SLX4 is implicated in mediating the interaction with the MUS81–EME1 complex to form a SLX–MUS HJ resolvase holoenzyme, but whether the SAP domain is involved in interaction with DNA is unknown. Second, although the SAP-CCD domain arrangement is conserved in SLX4 across species, the spacing between the two domains ranges from approximately a dozen residues in *S. cerevisiae* and *S. pombe* to more than a hundred residues in humans. The shortest human SLX4 fragment used in the study by Fekairi *et al.* contains ∼80 intervening residues in addition to the CCD domain. The extra residues may contain cryptic DNA binding motifs that can fulfill the role similar to the one played the SAP domain of yeast SLX4.

*In vitro*, the yeast SLX1–SLX4 complex efficiently cleaves 5′-flap DNA at the –3 position, but the efficiency and accuracy drop dramatically when the 5′-flap is only one nucleotide long. Our structure shows that the one nucleotide flap is wedged into the DNA duplex, rather than being freely disposed, and the insertion of the extra nucleotide distorts the local structure around the duplex-flap junction. The DNA is also kinked at the junction, and the –2 DNA backbone phosphate group is positioned next to the catalytic active site, instead of the –3 scissile phosphate for substrates with a longer 5′-flap, or the –5 scissile phosphate group with a 1-nt 5′flap, although the latter is much less efficient. These observations indicate that the DNA is trapped in a conformation resistant to nucleolytic removal of the 1-nt 5′-flap in the crystal structure. Furthermore, crystal packing may have stabilized the enzyme–DNA complex in an inactive state. Using DNA with a longer 5′-flap for MD simulation shows considerable conformational elasticity of the DNA substrate. Intriguingly, the longer 5′-flap in the MD model interacts with the positively charged surface region of SLX1 shown to bind DNA in the *Tt*SLX1–SLX4^CCD^-DNA structure, and the –2 phosphate, although still not the correct scissile phosphate, moved closer to the catalytic active site. Our mutagenesis results also identify the importance of this region for cleavage efficiency and specificity. Thus, a likely scenario for 5′-flap DNA processing by the SLX1–SLX4 complex may be as follows: the SAP domain of SLX4 binds the minor groove of DNA approximately one turn away from the flap-duplex junction in the post-nicked portion, and the 5′-flap engages the positively charged surface in the Uri domain of SLX1, in addition to the binding of α1 and the following loop to the major groove of DNA in the pre-nicked direction.

Our MD simulation is still unsatisfactory in one respect, namely, the DNA phosphodiester bond closest to the active site is between nucleotide –2 and –3, instead of the more prevalent cleavage site between nucleotide –3 and –4. We surmise that this is partly due to the bias introduced in the starting model, which is constructed using the double-stranded framework of DNA from the 1-nt 5′-flap DNA complex, where the DNA is distorted across the junction and one turn of DNA counts 11 basepairs. This starting model of the double-stranded portion of DNA appears to be trapped throughout the MD procedure. Two possible contributing factors of this bias maybe, first, the co-crystal structure we obtained is with the Y17F mutant. This change may affect the binding of a water molecule in the active site, indirectly perturbing DNA binding or destabilization of key residues nearby (Supplementary Figure S2A). In the structure of Hpy188I in complex with DNA, the corresponding tyrosine is seen to interact with the backbone phosphate directly or through a water molecule, depending whether it is in a product or substrate complex (38). Another possible factor is the involvement of metal ions, which is not included in our structure. Studies of FEN1 and other type of nucleases have revealed that metal ions could induce the shift of DNA for correct positioning of the cleavage site (43–45). We envision that mechanisms learned from studies of other type of nucleases, such as the involvement of metal ions and the sliding of the 5′-flap DNA, as implicated in FEN1 (42), could be operating in SLX1–SLX4 catalysis. It should be pointed out that, however, in addition to a main cleavage site, the yeast SLX1–SLX4 complex also cuts at several secondary sites *in vitro* (1). This property may be mitigated in the presence of other partners *in vivo* for distinct types of DNA structures. Nevertheless, further structural studies are needed to reveal precise mechanisms governing DNA cleavage site selection for different forms of DNA. We believe that our work on the structure and function of the SAP domain and the mechanism of 5′-flap recognition presented here represents a significant advance in the mechanistic understanding of the versatile SLX1–SLX4 complex.

## DATA AVAILABILITY

Atomic coordinates and associated structure factors for the structures reported here have been deposited in the Protein Data Bank with accession numbers 7CQ2, 7CQ3 and 7CQ4.

## Supplementary Material

gkab542_Supplemental_File

## References

[1] Fricke W.M. , Brill S.J. Slx1-Slx4 is a second structure-specific endonuclease functionally redundant with Sgs1-Top3. Genes Dev. 2003; 17:1768–1778. 12832395 10.1101/gad.1105203 PMC196184

[2] Mullen J.R. , Kaliraman V., Ibrahim S.S., Brill S.J. Requirement for three novel protein complexes in the absence of the Sgs1 DNA helicase in Saccharomyces cerevisiae. Genetics. 2001; 157:103–118. 11139495 10.1093/genetics/157.1.103 PMC1461486

[3] De Muyt A. , Jessop L., Kolar E., Sourirajan A., Chen J., Dayani Y., Lichten M. BLM helicase ortholog Sgs1 is a central regulator of meiotic recombination intermediate metabolism. Mol. Cell. 2012; 46:43–53. 22500736 10.1016/j.molcel.2012.02.020 PMC3328772

[4] Munoz I.M. , Hain K., Declais A.C., Gardiner M., Toh G.W., Sanchez-Pulido L., Heuckmann J.M., Toth R., Macartney T., Eppink B. et al. Coordination of structure-specific nucleases by human SLX4/BTBD12 is required for DNA repair. Mol. Cell. 2009; 35:116–127. 19595721 10.1016/j.molcel.2009.06.020

[5] Svendsen J.M. , Smogorzewska A., Sowa M.E., O’Connell B.C., Gygi S.P., Elledge S.J., Harper J.W. Mammalian BTBD12/SLX4 assembles a Holliday junction resolvase and is required for DNA repair. Cell. 2009; 138:63–77. 19596235 10.1016/j.cell.2009.06.030 PMC2720686

[6] Wyatt H.D. , Sarbajna S., Matos J., West S.C. Coordinated actions of SLX1–SLX4 and MUS81-EME1 for Holliday junction resolution in human cells. Mol. Cell. 2013; 52:234–247. 24076221 10.1016/j.molcel.2013.08.035

[7] Fekairi S. , Scaglione S., Chahwan C., Taylor E.R., Tissier A., Coulon S., Dong M.Q., Ruse C., Yates J.R., Russell P. et al. Human SLX4 is a Holliday junction resolvase subunit that binds multiple DNA repair/recombination endonucleases. Cell. 2009; 138:78–89. 19596236 10.1016/j.cell.2009.06.029 PMC2861413

[8] Coulon S. , Gaillard P.H.L., Chahwan C., McDonald W.H., Yates J.R., Russell P. Slx1-Slx4 are subunits of a structure-specific endonuclease that maintains ribosomal DNA in fission yeast. Mol. Biol. Cell. 2004; 15:71–80. 14528010 10.1091/mbc.E03-08-0586 PMC307528

[9] Coulon S. , Noguchi E., Noguchi C., Du L.L., Nakamura T.M., Russell P. Rad22Rad52-dependent repair of ribosomal DNA repeats cleaved by Slx1-Slx4 endonuclease. Mol. Biol. Cell. 2006; 17:2081–2090. 16467377 10.1091/mbc.E05-11-1006 PMC1415312

[10] Garner E. , Kim Y., Lach F.P., Kottemann M.C., Smogorzewska A. Human GEN1 and the SLX4-associated nucleases MUS81 and SLX1 are essential for the resolution of replication-induced Holliday junctions. Cell Rep. 2013; 5:207–215. 24080495 10.1016/j.celrep.2013.08.041 PMC3844290

[11] Rass U. Resolving branched DNA intermediates with structure-specific nucleases during replication in eukaryotes. Chromosoma. 2013; 122:499–515. 24008669 10.1007/s00412-013-0431-z PMC3827899

[12] Andersen S.L. , Bergstralh D.T., Kohl K.P., LaRocque J.R., Moore C.B., Sekelsky J. Drosophila MUS312 and the vertebrate ortholog BTBD12 interact with DNA structure-specific endonucleases in DNA repair and recombination. Mol. Cell. 2009; 35:128–135. 19595722 10.1016/j.molcel.2009.06.019 PMC2746756

[13] Wilson J.S. , Tejera A.M., Castor D., Toth R., Blasco M.A., Rouse J. Localization-dependent and -independent roles of SLX4 in regulating telomeres. Cell Rep. 2013; 4:853–860. 23994477 10.1016/j.celrep.2013.07.033 PMC3969258

[14] Zhang J. , Walter J.C. Mechanism and regulation of incisions during DNA interstrand cross-link repair. DNA Repair (Amst.). 2014; 19:135–142. 24768452 10.1016/j.dnarep.2014.03.018 PMC4076290

[15] Sarkar J. , Wan B., Yin J., Vallabhaneni H., Horvath K., Kulikowicz T., Bohr V.A., Zhang Y., Lei M., Liu Y. SLX4 contributes to telomere preservation and regulated processing of telomeric joint molecule intermediates. Nucleic Acids Res. 2015; 43:5912–5923. 25990736 10.1093/nar/gkv522 PMC4499145

[16] Saito T.T. , Mohideen F., Meyer K., Harper J.W., Colaiacovo M.P. SLX-1 is required for maintaining genomic integrity and promoting meiotic noncrossovers in the Caenorhabditis elegans germline. PLoS Genet. 2012; 8:e1002888. 22927825 10.1371/journal.pgen.1002888 PMC3426554

[17] Belfort M. , Roberts R.J. Homing endonucleases: keeping the house in order. Nucleic Acids Res. 1997; 25:3379–3388. 9254693 10.1093/nar/25.17.3379 PMC146926

[18] Suzuki R. , Shindo H., Tase A., Kikuchi Y., Shimizu M., Yamazaki T. Solution structures and DNA binding properties of the N-terminal SAP domains of SUMO E3 ligases from Saccharomyces cerevisiae and Oryza sativa. Proteins. 2009; 75:336–347. 18831036 10.1002/prot.22243

[19] Dodson C.A. , Ferguson N., Rutherford T.J., Johnson C.M., Fersht A.R. Engineering a two-helix bundle protein for folding studies. Protein Eng. Des. Sel. 2010; 23:357–364. 20130106 10.1093/protein/gzp080 PMC2851443

[20] Aravind L. , Koonin E.V. SAP - a putative DNA-binding motif involved in chromosomal organization. Trends Biochem. Sci. 2000; 25:112–114. 10694879 10.1016/s0968-0004(99)01537-6

[21] Castor D. , Nair N., Declais A.C., Lachaud C., Toth R., Macartney T.J., Lilley D.M., Arthur J.S., Rouse J. Cooperative control of holliday junction resolution and DNA repair by the SLX1 and MUS81-EME1 nucleases. Mol. Cell. 2013; 52:221–233. 24076219 10.1016/j.molcel.2013.08.036 PMC3808987

[22] Rouse J. Control of genome stability by SLX protein complexes. Biochem. Soc. Trans. 2009; 37:495–510. 19442243 10.1042/BST0370495

[23] Wan B. , Yin J., Horvath K., Sarkar J., Chen Y., Wu J., Wan K., Lu J., Gu P., Yu E.Y. et al. SLX4 assembles a telomere maintenance toolkit by bridging multiple endonucleases with telomeres. Cell Rep. 2013; 4:861–869. 24012755 10.1016/j.celrep.2013.08.017 PMC4334113

[24] Hodskinson M.R. , Silhan J., Crossan G.P., Garaycoechea J.I., Mukherjee S., Johnson C.M., Scharer O.D., Patel K.J. Mouse SLX4 is a tumor suppressor that stimulates the activity of the nuclease XPF-ERCC1 in DNA crosslink repair. Mol. Cell. 2014; 54:472–484. 24726326 10.1016/j.molcel.2014.03.014 PMC4017094

[25] Klein Douwel D. , Boonen R.A., Long D.T., Szypowska A.A., Raschle M., Walter J.C., Knipscheer P. XPF-ERCC1 acts in unhooking DNA interstrand crosslinks in cooperation with FANCD2 and FANCP/SLX4. Mol. Cell. 2014; 54:460–471. 24726325 10.1016/j.molcel.2014.03.015 PMC5067070

[26] Gaur V. , Wyatt H.D.M., Komorowska W., Szczepanowski R.H., de Sanctis D., Gorecka K.M., West S.C., Nowotny M. Structural and mechanistic analysis of the Slx1-Slx4 endonuclease. Cell Rep. 2015; 10:1467–1476. 25753413 10.1016/j.celrep.2015.02.019 PMC4407285

[27] Gaur V. , Ziajko W., Nirwal S., Szlachcic A., Gapinska M., Nowotny M. Recognition and processing of branched DNA substrates by Slx1-Slx4 nuclease. Nucleic Acids Res. 2019; 47:11681–11690. 31584081 10.1093/nar/gkz842 PMC6902002

[28] Otwinowski Z. , Minor W. Processing of X-ray diffraction data collected in oscillation mode. Methods Enzymol. 1997; 276:307–326. 10.1016/S0076-6879(97)76066-X 27754618

[29] Sheldrick G.M. A short history of SHELX. Acta Crystallogr. A. 2008; 64:112–122. 18156677 10.1107/S0108767307043930

[30] Adams P.D. , Afonine P.V., Bunkoczi G., Chen V.B., Davis I.W., Echols N., Headd J.J., Hung L.W., Kapral G.J., Grosse-Kunstleve R.W. et al. PHENIX: a comprehensive Python-based system for macromolecular structure solution. Acta Crystallogr. D. Biol. Crystallogr. 2010; 66:213–221. 20124702 10.1107/S0907444909052925 PMC2815670

[31] Emsley P. , Cowtan K. Coot: model-building tools for molecular graphics. Acta Crystallogr. D. Biol. Crystallogr. 2004; 60:2126–2132. 15572765 10.1107/S0907444904019158

[32] Vagin A. , Teplyakov A. Molecular replacement with MOLREP. Acta Crystallogr. D. Biol. Crystallogr. 2010; 66:22–25. 20057045 10.1107/S0907444909042589

[33] Zhou T. , Xiong J., Wang M., Yang N., Wong J., Zhu B., Xu R.M. Structural basis for hydroxymethylcytosine recognition by the SRA domain of UHRF2. Mol. Cell. 2014; 54:879–886. 24813944 10.1016/j.molcel.2014.04.003

[34] Waterhouse A. , Bertoni M., Bienert S., Studer G., Tauriello G., Gumienny R., Heer F.T., de Beer T.A.P., Rempfer C., Bordoli L. et al. SWISS-MODEL: homology modelling of protein structures and complexes. Nucleic Acids Res. 2018; 46:W296–W303. 29788355 10.1093/nar/gky427 PMC6030848

[35] Anandakrishnan R. , Aguilar B., Onufriev A.V. H++ 3.0: automating pK prediction and the preparation of biomolecular structures for atomistic molecular modeling and simulations. Nucleic Acids Res. 2012; 40:W537–W541. 22570416 10.1093/nar/gks375 PMC3394296

[36] Maier J.A. , Martinez C., Kasavajhala K., Wickstrom L., Hauser K.E., Simmerling C. ff14SB: improving the accuracy of protein side chain and backbone parameters from ff99SB. J. Chem. Theory Comput. 2015; 11:3696–3713. 26574453 10.1021/acs.jctc.5b00255 PMC4821407

[37] Ivani I. , Dans P.D., Noy A., Perez A., Faustino I., Hospital A., Walther J., Andrio P., Goni R., Balaceanu A. et al. Parmbsc1: a refined force field for DNA simulations. Nat. Methods. 2016; 13:55–58. 26569599 10.1038/nmeth.3658 PMC4700514

[38] Sokolowska M. , Czapinska H., Bochtler M. Hpy188I-DNA pre- and post-cleavage complexes–snapshots of the GIY-YIG nuclease mediated catalysis. Nucleic Acids Res. 2011; 39:1554–1564. 20935048 10.1093/nar/gkq821 PMC3045582

[39] Dunin-Horkawicz S. , Feder M., Bujnicki J.M. Phylogenomic analysis of the GIY-YIG nuclease superfamily. BMC Genomics. 2006; 7:98. 16646971 10.1186/1471-2164-7-98 PMC1564403

[40] Truglio J.J. , Rhau B., Croteau D.L., Wang L., Skorvaga M., Karakas E., DellaVecchia M.J., Wang H., Van Houten B., Kisker C. Structural insights into the first incision reaction during nucleotide excision repair. EMBO J. 2005; 24:885–894. 15692561 10.1038/sj.emboj.7600568 PMC554121

[41] Mak A.N. , Lambert A.R., Stoddard B.L. Folding, DNA recognition, and function of GIY-YIG endonucleases: crystal structures of R.Eco29kI. Structure. 2010; 18:1321–1331. 20800503 10.1016/j.str.2010.07.006 PMC2955809

[42] Grasby J.A. , Finger L.D., Tsutakawa S.E., Atack J.M., Tainer J.A. Unpairing and gating: sequence-independent substrate recognition by FEN superfamily nucleases. Trends Biochem. Sci. 2012; 37:74–84. 22118811 10.1016/j.tibs.2011.10.003 PMC3341984

[43] Tsutakawa S.E. , Thompson M.J., Arvai A.S., Neil A.J., Shaw S.J., Algasaier S.I., Kim J.C., Finger L.D., Jardine E., Gotham V.J.B. et al. Phosphate steering by Flap Endonuclease 1 promotes 5'-flap specificity and incision to prevent genome instability. Nat. Commun. 2017; 8:15855. 28653660 10.1038/ncomms15855 PMC5490271

[44] Nowotny M. , Yang W. Stepwise analyses of metal ions in RNase H catalysis from substrate destabilization to product release. EMBO J. 2006; 25:1924–1933. 16601679 10.1038/sj.emboj.7601076 PMC1456928

[45] Shi Y. , Hellinga H.W., Beese L.S. Interplay of catalysis, fidelity, threading, and processivity in the exo- and endonucleolytic reactions of human exonuclease I. Proc. Natl. Acad. Sci. U.S.A. 2017; 114:6010–6015. 28533382 10.1073/pnas.1704845114 PMC5468604

