# Supplementary material for: Structure specific DNA recognition by the SLX1–SLX4 endonuclease complex
Source: Nucleic Acids Res. 2021 Jun 28;49(13):7740–52. doi: 10.1093/nar/gkab542 (PMC8287910; doi:10.1093/nar/gkab542)
Supplement: gkab542_Supplemental_File [file gkab542_supplemental_file.pdf]

SUPPLEMENTARY INFORMATION

Structure specific DNA recognition by the SLX1-SLX4 endonuclease complex

Xiang Xu[1,2,#], Mingzhu Wang[3,#], Jixue Sun[1,#], Zhenyu Yu[2,#], Guohong Li[2,4], Na Yang[1,*], and Rui-Ming Xu[2,4,*]

[1] State Key Laboratory of Medicinal Chemical Biology, College of Pharmacy and Key Laboratory of Medical Data Analysis and Statistical Research of Tianjin, Nankai University, Tianjin 300353, China;

[2] National Laboratory of Biomacromolecules, CAS Center for Excellence in Biomacromolecules, Institute of Biophysics, Chinese Academy of Sciences, Beijing 100101, China;

[3] Institutes of Physical Science and Information Technology, Anhui University, Hefei 230601, Anhui, China;

[4] University of Chinese Academy of Sciences, Beijing 100049, China;

[#] Co-first authors.

[*] Correspondence: yangnanku@nankai.edu.cn; rmxu@ibp.ac.cn

Table S1. DNA sequences and structures

DNA	Usage	Sequences	Structure after Annealing
1-nt<br>5'-flap	Crystallization with Slx1-Slx4[SAP+CCD]	5'-AGGACATCTTTGCC-3'<br>5'-CGGCAAAGATGTCC<br>ATCTGTTGTAATC-3'<br>5'-GGATTACAACAGAT-3'	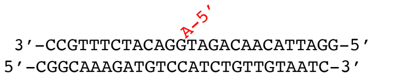
1-nt<br>5'-flap	Nuclease assay	5'-AGGACATCTTTGCC-Cy3<br>5'-CGGCAAAGATGTCC<br>ATCTGTTGTAATC-3'<br>5'-GGATTACAACAGAT-3'	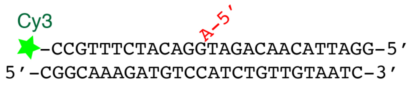
5'-flap	Nuclease assay	XO-1:<br>Cy3-ACCAGTGCCTTGCTA<br>GGACATCTTTGCCCA-3'<br>XO-2:<br>5'-TGGGCAAAGATGTCC<br>ATCTGTTGTAATCGT-3'<br>XO-3a:<br>5'-ACGATTACAACAGAT-3'	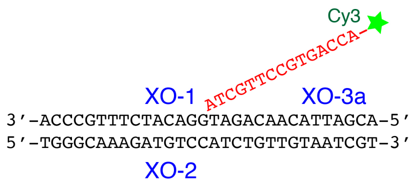
HJ	Nuclease assay	XO-1, XO-2, and<br>XO-3:<br>5'-ACGATTACAACAGAT<br>CATGGAGCTGTCTAG-3'<br>XO-4:<br>5'-CTAGACAGCTCCATG<br>TAGCAAGGCACTGGT-3'	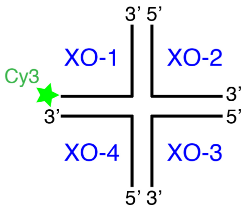

Figure S1. 2.5 Å crystal structure of the SLX1-SLX4$^{SAP+CCD}$ complex

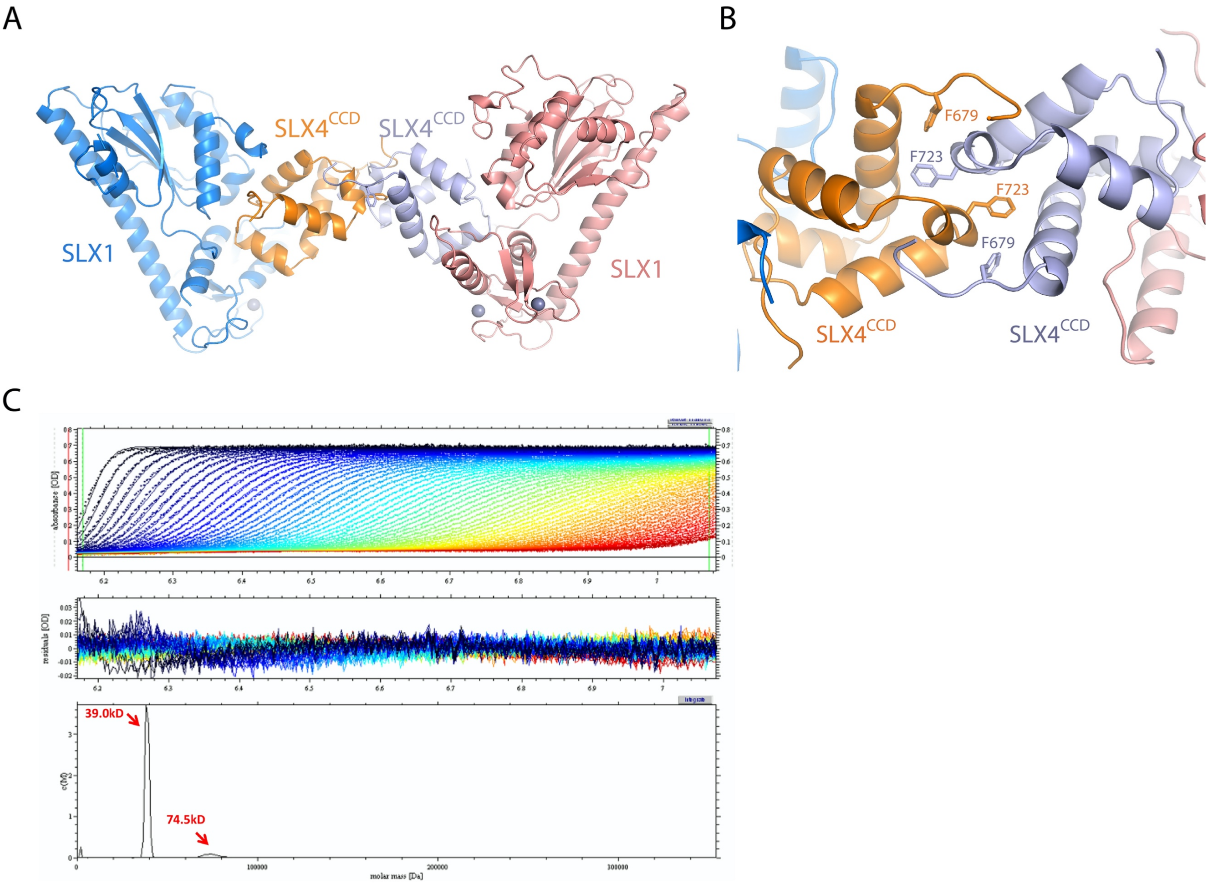

(A) Two SLX1-SLX4$^{SAP+CCD}$ complexes in one asymmetric unit of the $P2_12_12_1$ crystal. SLX1 molecules are colored marine and salmon, and the CCD domains of SLX4 are colored orange and light blue, respectively, while the SAP domain is disordered. (B) Interactions between the two CCD domains of SLX4, notably through F679 and F723 from each CCD domain, in the asymmetric unit. The inter-domain interaction involves an interface area of 1014 Å^2. (C) The heterodimer-heterodimer interaction is likely a crystallization artifact, as an analytic ultracentrifugation analysis reveals that the majority of the proteins exists as a smaller complex in solution, as indicated in the bottom panel. The theoretical value for the SLX1-SLX4$^{SAP+CCD}$ heterodimer is 51.6 kD, and that for SLX1-SLX4^{CCD} is 44.5 kD. The concentration of the protein sample was at OD_{280}=0.82, and the velocity sedimentation experiment was performed using a ProteomeLab XL-I (Beckman Coulter) device at 60,000g for 6 h. The raw data are displayed in the top panels, and the molecular mass derived from the calculated sedimentation coefficients using the program Sedfit is shown at the bottom panel.

Figure S2. Comparison of *S. cerevisiae* SLX1-SLX4^{CCD} structures

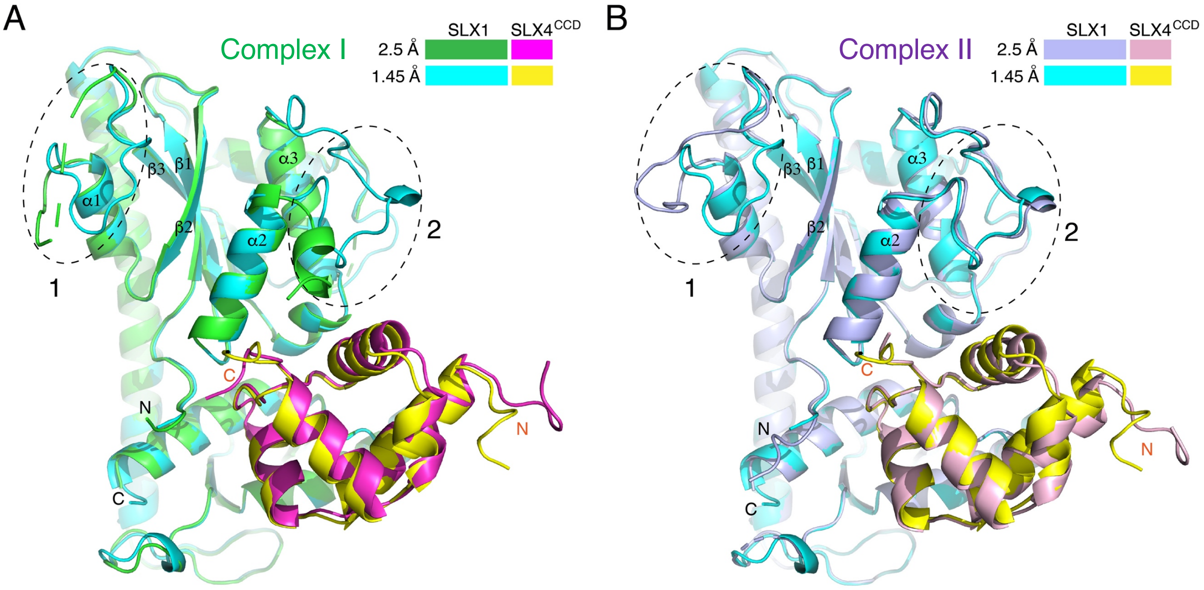

(A)-(B) Superposition of the 1.45 Å, $P2_12_12$ structure with each (Complex I and II) of the two SLX1-SLX4^{CCD} heterodimers in the 2.5 Å, $P2_12_12_1$ structure. SLX1 and SLX4 are colored cyan and yellow for the 1.45 Å structure, green and magenta for Complex I, and light blue and light pink for Complex II, respectively. The RMSD values are 0.45 Å and 0.72 Å between SLX1 from the 1.45 Å structure and that of Complex I and II, respectively, using 261 and 281 residues of SLX1 for alignment. Two regions showing the most differences are indicated with dashed-line ellipses: region 1 includes the loop between α1 and β3 (a.a. 46-57), and region 2 spans the loop connecting α2 and α3 (a.a. 88-106).

Figure S3. Alignment of SLX1 and SLX4 sequences

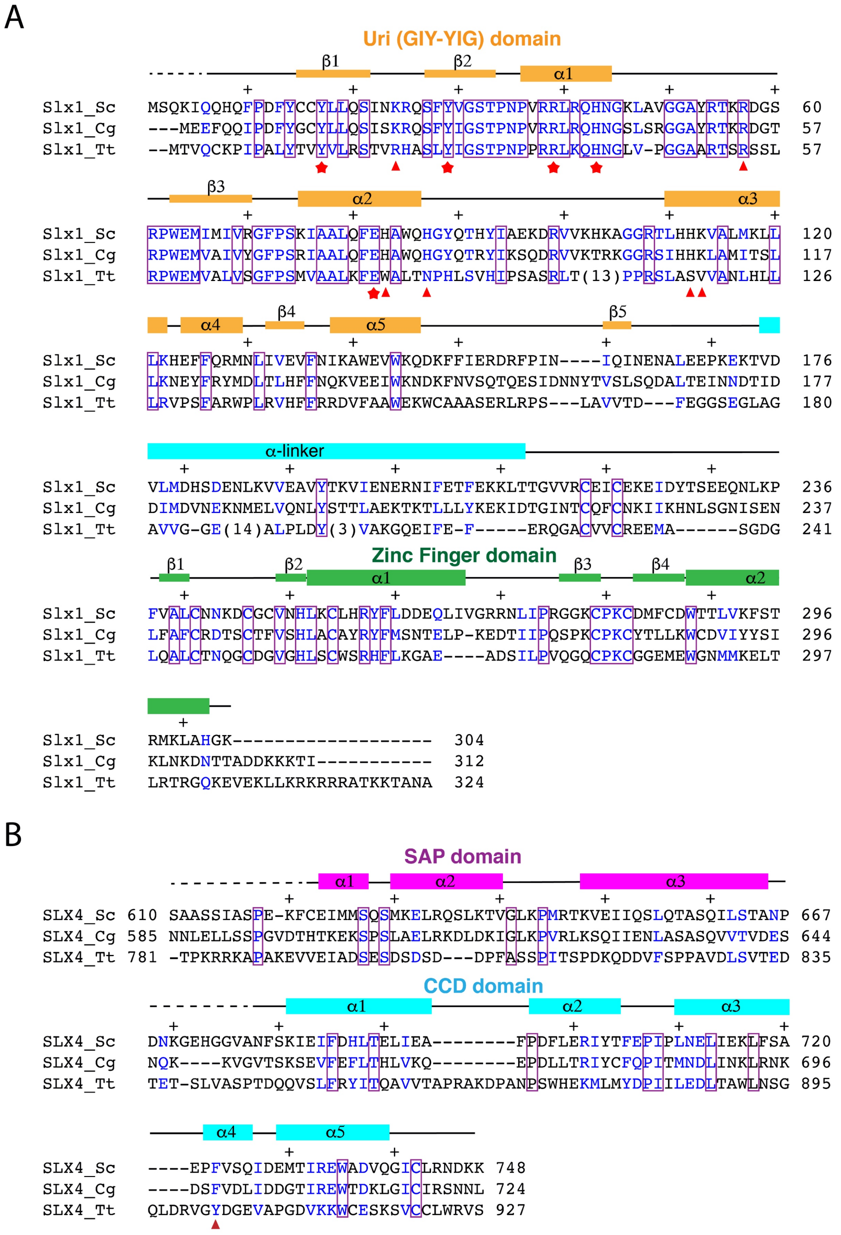

(A) Alignment of amino acid sequences of SLX1 from *S. cerevisiae* (Sc), *C. glabrata* (Cg) and *T. terrestris* (Tt), with UniProt IDs of B3LMT5, Q6FML9, and G2QV68, respectively. Identical and similar residues are shown in blue letters, and the former is also enclosed in purple line boxes. The numbers at the end of each line number the nearby residue, and the "+" signs at the top of the sequence mark every tenth residues of *Sc*SLX1. Secondary structure elements based on the *Sc*SLX1 structure are displayed at the top of the sequence, colored according to the labelled protein domains. Red asterisks mark the active site residues, and red triangles indicate the residues subjected to mutational analyses. (B) Alignment of the SLX4 fragments, starting from residues numbered at the beginning of the sequences of *Sc*, *Cg* and *Tt* SLX4 (UniProt IDs Q12098, Q6FJQ6, G2RCY5, respectively). The alignment is labelled and displayed following the same rules as described above.

Figure S4. Comparison of SLX1-SLX4^{CCD} structures from different species

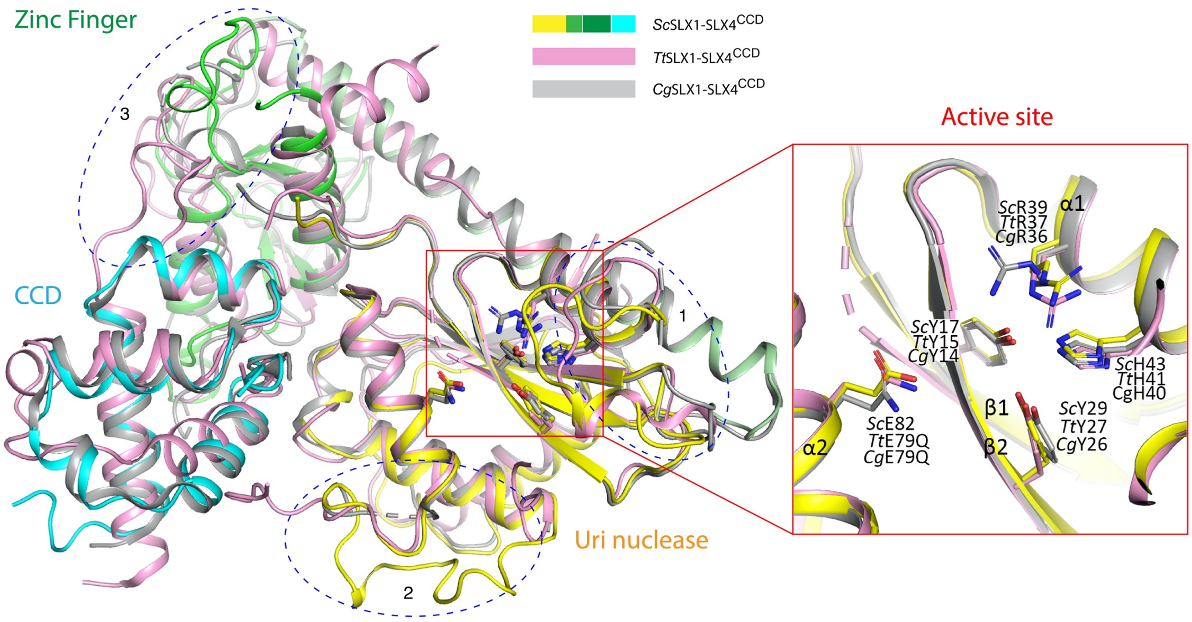

Superposition of the SLX1-SLX4^{CCD} structures from *S. cerevisiae* (*Sc*), *C. glabrata* (*Cg*, PDB ID: 4XLG) and *T. terrestris* (*Tt*, PDB ID: 6SEI), colored following the scheme displayed on the filled rectangles. The protein domains are labelled. The most variable regions among the three structures are indicated with dashed-line ellipses and labelled from 1 to 3. Regions 1 and 2 are the same areas that differ between *Sc*SLX1 structures crystallized in different forms, as described in Figure S2. Additionally, region 3 in the zinc finger motif shows considerable degrees of structural variations. The area boxed with red lines indicate the active site, where the five invariant catalytic residues are shown in a stick representation. The inset shows an enlarged view of superposition of the active site residues.

Figure S5. The SAP domain of SLX4 has intrinsic DNA binding property

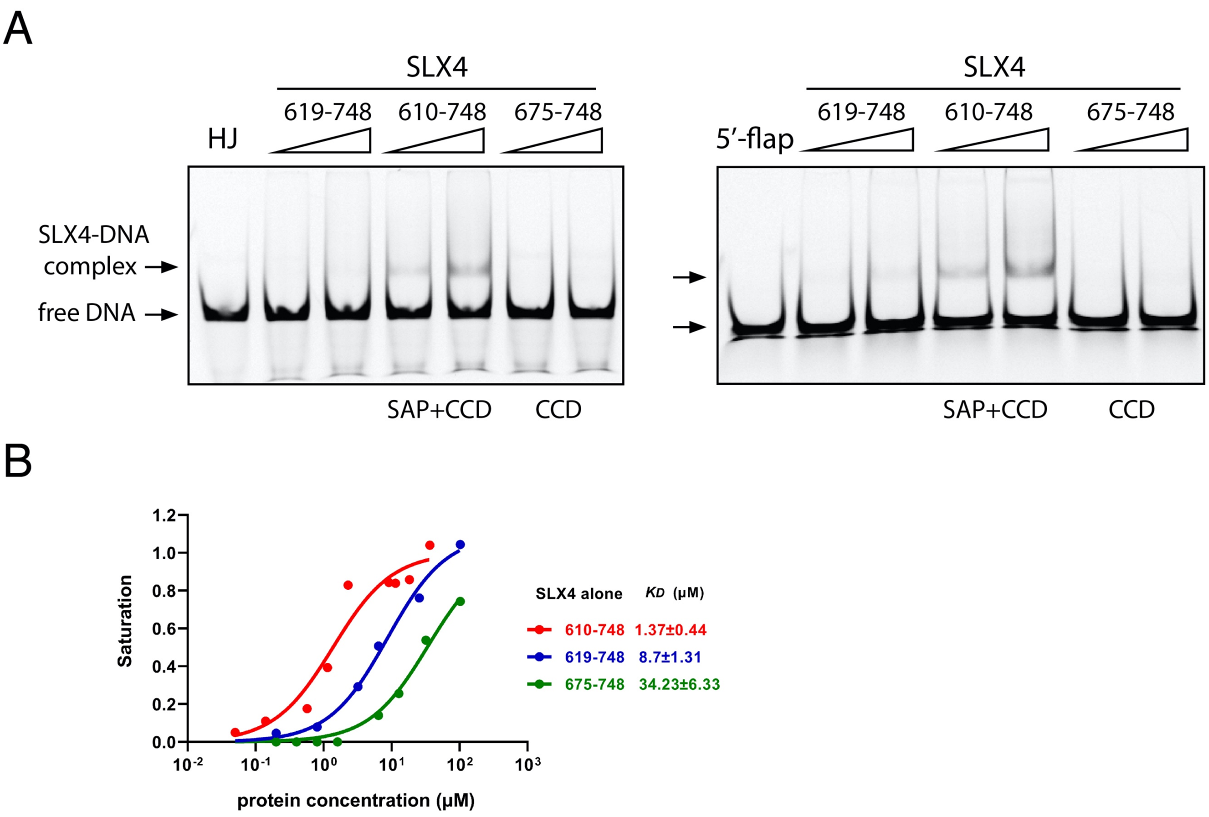

(A) EMSA with Holiday Junction (left panel) and 5'-flap DNA (right panel) with indicated SLX4 fragments at 30:1 and 60:1 protein to DNA molar ratios shows that the SAP+CCD domain of SLX4 (a.a. 610-748) can bind DNA on its own, while the CCD domain (a.a. 675-748) binds DNA below the detection level. (B) Fluorescence Polarization Assay (FPA) of 5'-flap DNA binding by the indicated SLX4 fragments. The derived K_D values corresponding to each of the SLX4 fragments are displayed at the right.

Figure S6. Structure of SLX1-SLX4$^{SAP+CCD}$ in complex with DNA

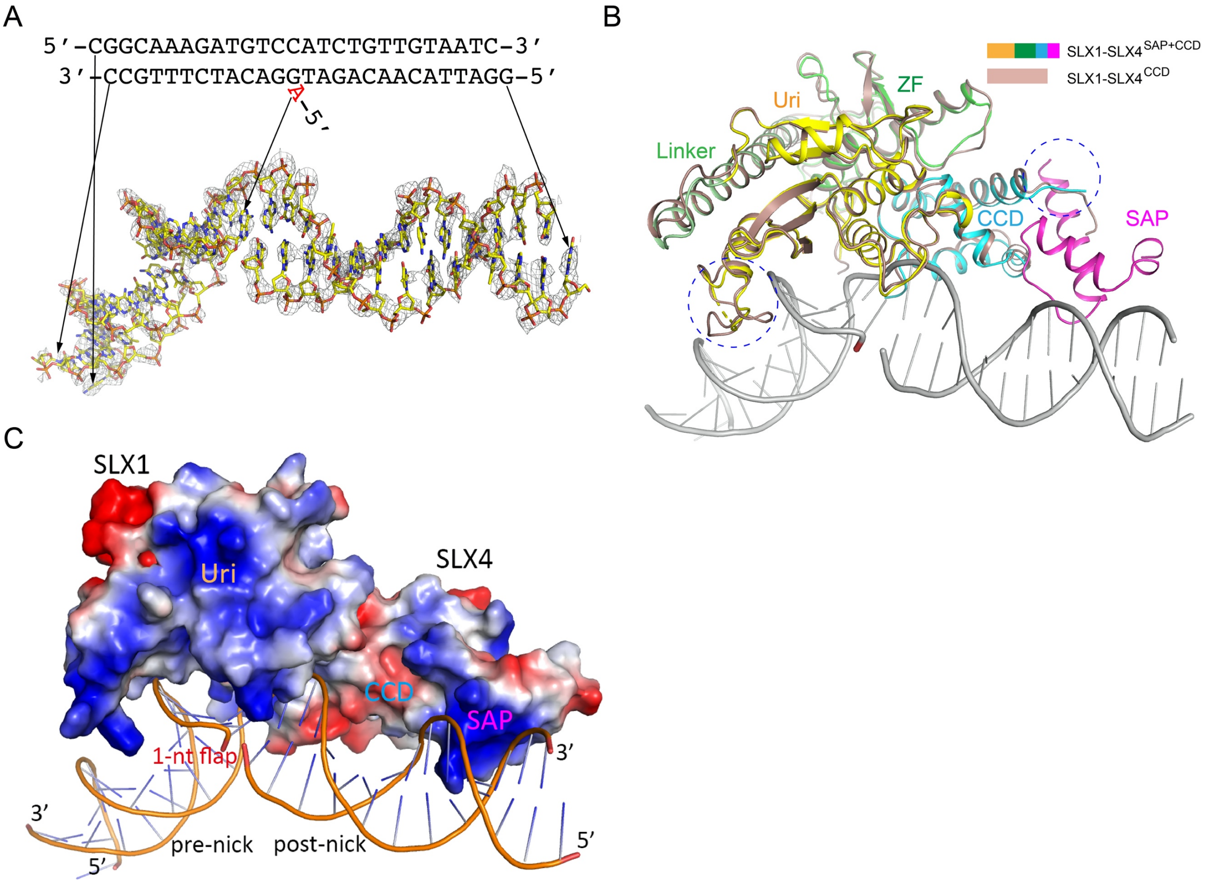

(A) Simulated annealing omit electron density map of the 1-nt 5'-flap DNA. The map was generated with the DNA not included in the refinement, and the Fo-Fc map was contoured at 2σ level. The DNA sequence is shown at the bottom, with the 5'-flap Adenine colored red and pointed to the corresponding base in the omit map with an arrow head. Terminal nucleotides are also indicated with arrow heads. (B) Superposition of the SLX1-SLX4$^{SAP+CCD}$-DNA structure (multicolored domains) with the apo SLX1-SLX4^{CCD} structure (beige). The dashed-line circles indicate gap regions in the structure. (C) Electrostatic potential distribution (positive, blue; negative, red; neutral, white) on the protein surface of the SLX1-SLX4$^{SAP+CCD}$-DNA complex.

Figure S7. Distinct DNA binding regions of the SLX1-SLX4 complex

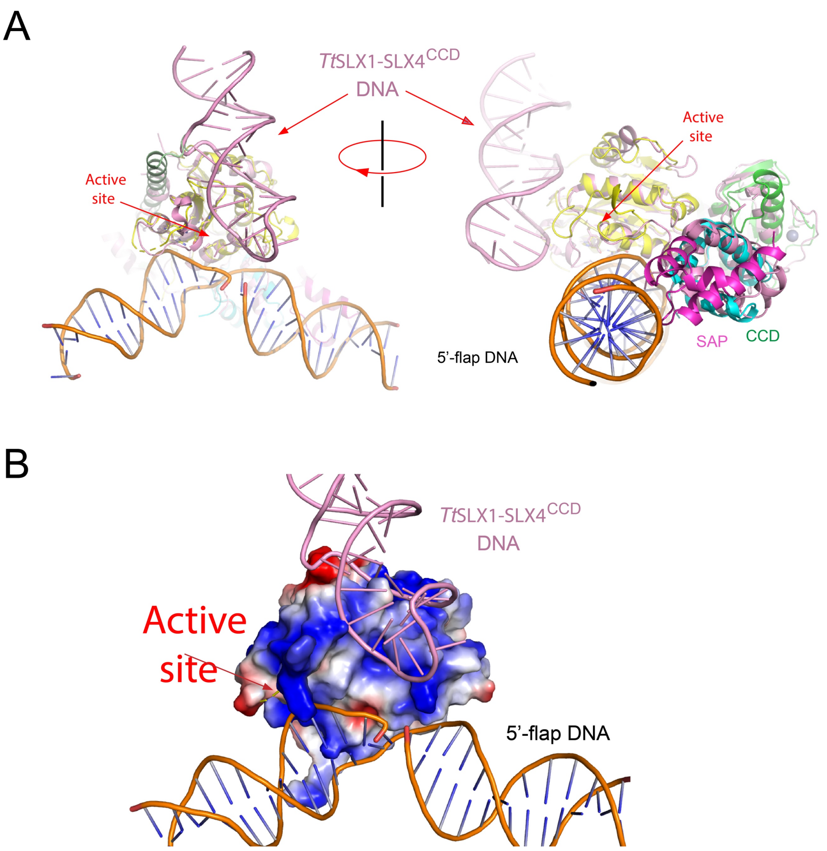

(A) Superposition of the DNA (pink) from the *T. terrestris* SLX1-SLX4^{CCD}-DNA complex (PDB ID: 6SEI) on to the *S. cerevisiae* SLX1-SLX4$^{SAP+CCD}$-DNA structure shows that the DNA is bound to the side of Uri nuclease domain distinct from where the 5'-flap DNA binds. (B) The location of the T. terrestris DNA binding site corresponds to a positively charged surface region of the S. cerevisiae SLX1. SLX4 domains are not shown for viewing clarity. The location of the catalytic active site is indicated.

Figure S8. Comparison of 5’-flap DNA structures

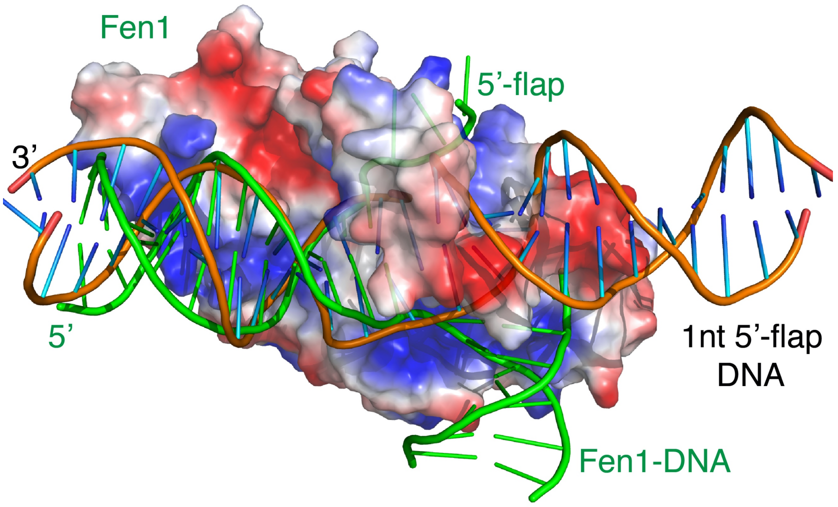

The 1-nt 5’-flap DNA from the structure with SLX1-SLX4$^{SAP+CCD}$, shown in a cartoon representation and colored in orange, is superimposed with the 5’-flap DNA substrate, colored in green, of Flap Endonuclease 1 (FEN1, PDB ID: 5KSE), aligned using the first three basepairs in the pre-nick portion from the flap junction by visual inspection. FEN1 is shown with a semi-transparent protein electrostatic potential surface representation. The 5’-flap of the FEN1 substrate is bound capped gateway, and post-nick stem is almost perpendicular to the pre-nick stem, and the pre-nick portion of superimposed 1-nt 5’-flap DNA from the structure complexed with SLX1-SLX4$^{SAP+CCD}$.

Figure S9. Crystal packing stabilizes DNA conformation in the SLX1-SLX4$^{SAP+CCD}$-DNA complex

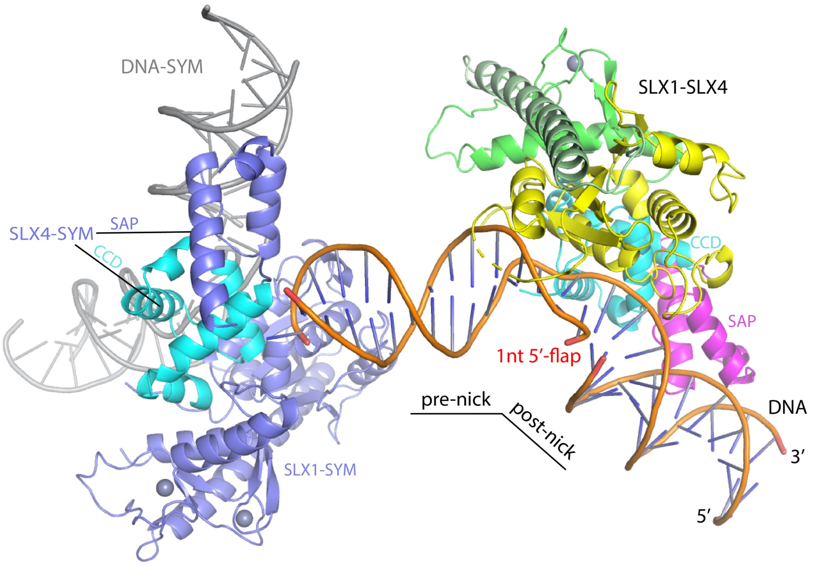

The pre-nick end of the 1nt 5'-flap DNA is involved in the crystal packing a symmetry-related SLX1 in a neighboring asymmetric unit. This interaction stabilizes the DNA conformation, and may contribute to the distortion of the pre-nick portion of DNA in the crystal structure.

Figure S10. Modeling 5'-flap DNA substrate binding to the SLX1-SLX4$^{SAP+CCD}$ complex

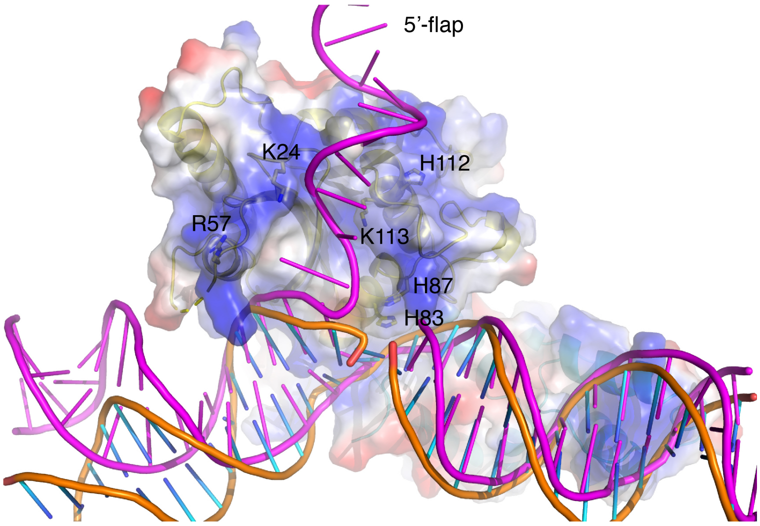

DNA substrate with a 10-nt 5'-flap based on a 1-μs MD simulation (magenta) is superimposed with the crystal structure of SLX1-SLX4$^{SAP+CCD}$-DNA complex. The protein complex was shown in a semi-transparent electrostatic potential surface representation, superimposed with a cartoon diagram of the proteins, with SLX1 and SLX4 colored in yellow and cyan, respectively. Several SLX1 residues situated in the positively charged surface area next to the modeled 5'-flap binding cleft are shown in a stick representation and labeled.
